# Development of UPLC method for simultaneous assay of some COVID-19 drugs utilizing novel instrumental standard addition and factorial design

**DOI:** 10.1038/s41598-023-32405-x

**Published:** 2023-04-04

**Authors:** Hanan I. El-Shorbagy, Mona A. Mohamed, Alaa El-Gindy, Ghada M. Hadad, Fathalla Belal

**Affiliations:** 1 Pharmaceutical Analytical Chemistry Department, Faculty of Pharmacy, Suez Canal University, Ismailia, 41522 Egypt; 2 Pharmaceutical Chemistry Department, Egyptian Drug Authority (EDA), Cairo, Egypt; 3 Department of Pharmaceutical Analytical Chemistry, Faculty of Pharmacy, Mansoura University, Mansoura, 35516 Egypt

**Keywords:** Analytical chemistry, Green chemistry, Drug development

## Abstract

A green, rapid, and simple RP-UPLC method was developed and optimized by full factorial design for the simultaneous separation of oseltamivir phosphate, daclatasivir dihydrochloride, and remdesivir, with dexamethasone as a co-administered drug. The separation was established on a UPLC column BEH C_18_ 1.7 µm (2.1 × 100.0 mm) connected with a UPLC pre-column BEH 1.7 µm (2.1 × 5.0 mm) at 25 °C with an injection volume of 10 µL. The detector (PDA) was set at 239 nm. The mobile phase consisted of methanol and ammonium acetate (8.1818 mM) in a ratio of 75.7: 24.3 (v/v). The flow rate was set at 0.048 mL min^−1^. The overall separation time was 9.5 min. The retention times of oseltamivir phosphate, dexamethasone, daclatasivir dihydrochloride, and remdesivir were 6.323 ± 0.145, 7.166 ± 0.036, 8.078 ± 0.124, and 8.572 ± 0.166 min (eight replicates), respectively. The proposed method demonstrated linearity in the ranges of 10.0–500.0 (ng mL^−1^) and 0.5–30.0 (µg mL^−1^) for oseltamivir phosphate, 50.0–5000.0 (ng mL^−1^) for dexamethasone, 25.0–1000.0 (ng mL^−1^) and 0.5–25.0 (µg mL^−1^) for daclatasvir dihydrochlorde, and 10.0–500.0 (ng mL^−1^) and 0.5–30.0 (µg mL^−1^) for remdesivir. The coefficients of determination (R^2^) were greater than 0.9999, with percentage recoveries greater than 99.5% for each drug. The limits of quantitation were 6.4, 1.8, 7.8, and 1.6 ng mL^−1^, and the limits of detection were 1.9, 0.5, 2.0, and 0.5 ng mL^−1^ for oseltamivir phosphate, dexamethasone, daclatasivir dihydrochloride, and remdesivir, respectively. The proposed method was highly precise, as indicated by the low percentage of relative standard deviation values of less than 1.2% for each drug. The average content and uniformity of dosage units in the studied drugs' dosage forms were determined. The average contents of oseltamivir phosphate, dexamethasone, daclatasivir dihydrochloride, and remdesivir were nearly 93%, 102%, 99%, and 95%, respectively, while the uniformity of dosage unit values were nearly 92%, 102%, 101%, and 97%. Two novel methods were established in this work. The first method was used to assess the stability of standard solutions. This novel method was based on the slope of regression equations. The second was to evaluate the excipient's interference using an innovative instrumental standard addition method. The novel instrumental standard addition method was performed using the UPLC instrument program. It was more accurate, sensitive, time-saving, economical, and eco-friendly than the classic standard addition method. The results showed that the proposed method can estimate the tested drugs' concentrations without interference from their dosage form excipients. According to the Eco-score (more than 75), the Green Analytical Procedure Index (GAPI), and the AGREE criteria (total score of 0.77), the suggested method was considered eco-friendly.

## Introduction

COVID-19 is considered a severe problem for healthcare systems and economies, as well as vulnerable populations such as the elderly and those with chronic health conditions. COVID-19 was caused by the severe acute respiratory syndrome coronavirus 2 (SARS-CoV-2). Corona viruses are enclosed viruses with a positive-polarity single-stranded RNA genome. Because of this lethal sickness, researchers worldwide are forced to produce appropriate medicines for its prevention and treatment. Oseltamivir phosphate (OSTP) (Fig. S1A), dexamethasone (DEX) (Fig. S1B), daclatasivir dihydrochloride (DAC) (Fig. S1C), and remdesivir (REM) (Fig. S1D) were reported for SARS-CoV-2 infection^1–4^.

OSTP is a neuraminidase inhibitor that inhibits the viral neuraminidase enzyme present on the virus' surface. This action prevents the budding of the virus from the host cell, replicating, and infecting. It is used to treat and prevent infections with influenza viruses A (including pandemic H1N1) and B. It was approved by the U.S. Food and Drug Administration (FDA) in August 2016. DEX is a glucocorticoid. It is used for the treatment of inflammatory diseases. It was approved by the FDA in October 1958. It is used for COVID-19 patients with severe respiratory symptoms and is given in conjunction with other antiviral medications. DAC is a direct-acting antiviral drug. It was approved by the FDA in July 2015. It is used in conjunction with other antiviral medications to treat hepatitis C virus (HCV) infections. The daclatasivir/sofosbuvir combination was observed to enhance clinical outcomes in individuals who had moderate-to-severe COVID-19 symptoms^1^. REM is a nucleoside analog. It prevents viral replication by inhibiting the RNA-dependent RNA polymerase enzyme. In October 2020, the FDA approved it for the treatment of COVID-19.

Several analytical methods for the determination of OSTP, DEX, DAC, and REM in dosage forms and plasma have been documented in the literature. According to literature studies, OSTP was determined by chromatographic, capillary zone electrophoresis (CE), enzymatic, spectrofluorimetric, mass spectrometric (MS), colorimetric, and spectrophotometric techniques since it was approved by FDA until 2011^5,6^. Since 2011, OSTP has been determined by UPLC^7^, RP-HPLC^8^, LC–MS/MS^9^, potentiometric^10^, spectrofluorimetric^11^, and spectrophotometric^12^ methods. OSTP in combination with compounds other than DEX, DAC, and REM was determined by RP-HPLC^13,14^, LC–MS/MS^15^, and mass spectrometric (MS)^16^. DEX has been determined by RP-HPLC-UV, UPLC-UV, LC–MS/MS, and spectrophotometric methods since it was approved by FDA until 2022^17^. DAC has been determined by RP-HPLC^18^, UPLC^18^, spectrofluorimetric^19^, spectrophotometric^20^ techniques since it was approved by FDA in 2015,. DAC in combination with compounds other than OSTP, DEX, and REM was determined by RP-HPLC^21,22^, electrochemical^23^, spectrofluorimetric^24^, and CE^25^ techniques. REM has been determined by RP-HPLC^26^, LC–MS/MS^27^, spectrofluorimetric^28^, and electrochemical^23^ techniques. REM with DEX has been determined by the UPLC^29^ method. No analytical method was reported to estimate OSTP, DEX, DAC, and REM simultaneously.

During the development of the proposed method, two challenges were anticipated. The first was due to the high degree of ionization of DAC and OSTP, so they would create tailed peaks. The second was peak overlapping between OSTP and DEX and between DAC and REM because of their close log P values. The aim of this work was to develop and validate a green, simple, highly-sensitive, fast, and accurate RP-UPLC-PDA technique for the simultaneous determination of OSTP, DEX, DAC, and REM without interference from their dosage form excipients.

The proposed method was optimized using the design of experiments (DOE) technique^30^. DOE is a statistics-based technique. It has numerous benefits in optimization and the development of a robust method^31^. DOE optimization requires significantly less investment, effort, and resources than univariate procedures^32^. Because of the DOE's research, it is now possible to understand and improve the method's performance by identifying key method parameters and designing plots that ensure the method's stability and best performance^33^. The robustness and system suitability parameters of the developed method were estimated using mathematical equations. These mathematical equations were derived from the estimated coefficients (in uncoded units), which were estimated by the full factorial design (2^3^ FFD)^31,34^.

Two novel methods were established in this work. The first method was used to assess the stability of standard solutions. This novel method was based on the slope of regression equations. The second was to evaluate the excipient's interference using an innovative instrumental standard addition method. The novel instrumental standard addition method was performed using the UPLC instrument program. The novel instrumental standard addition method was more accurate, sensitive, time-saving, economical, and eco-friendly than the classic standard addition method. The results showed that the proposed method can estimate OSTP, DEX, DAC, and REM without interference from their dosage form excipients.

According to BP^35^, the suggested method was used to calculate the average content and uniformity of dosage unit. The results were compared to previously published RP-HPLC-UV methods^8,22,26,36^. The suggested method will be useful for quality control evaluation of the pharmaceuticals under investigation in their dosage units, as well as for future polypill formulations. The proposed method is appropriate for UV detectors (PDA at one wavelength) and LC–MS/MS method development since the mobile phases utilized (methanol and ammonium acetate) are appropriate for mass detectors. The proposed method is simple in sample preparation. In addition, Eco-Scale, the Green Analytical Procedure Index (GAPI) guidelines, and AGREE (Analytical GREEnness) analysis were used to assess the suggested method's greenness^37–39^. The proposed method is a more eco-friendly, time-saving, and cost-saving method than the reported methods^8,22,26,36^.

## Experimental

### Apparatus and software

The developed method was carried out on an ACQUITY UPLC H-Class PLUS System (U S A) outfitted with a WATERS quaternary solvent manager (M20QSP 471A), a WATERS column heater compartment (K20CHA 249G) with an ACQUITY Waters UPLC column BEH C_18_ 1.7-µm (2.1 $\mathrm{X}$ 100 mm) connected to an ACQUITY Waters UPLC pre-column BEH 1.7-µm VanGuard (2.1 $\mathrm{X}$ 5 mm), a WATERS temperature controlled sample manager-flow through needle (SM-FTN-H) with a volume range of 0.1 to 1000 μL (L20FTP 311G), and an ACQUITY photodiode array (PDA) eλ detector (L20UPL 182A). WATERS Empower3 chromatography software was used to record and analyze the data.

Statistical analysis of the factorial design was performed using MINITAB statistical software (release 16, State College, Pennsylvania).

Water deionizer (Stakpure-OmniaLab, Germany), calibrated sensitive balance (Sartorius, Germany), pH meter (Jenway 3510, UK), ultrasonic (Elma, Germany), mixer (WiseMix, Portugal), oil-free vacuum pump (Rocker 400, Taiwan), and nylon membrane filters (47-mm in diameter, 0.22-mm pore size, Chromtech, UK) were used for mobile phase preparation. Pipet4u pro manual pipettes 2–20, 10–100, 20–200, and 100–1000 µL (Biotechnologie GmbH, Germany) were used for the preparation of standard and sample solutions. Syringe filters (25 and 13 mm in diameter, 0.22 m pore size, Chromtech PTFE, UK) were used to filtrate standard and sample solutions before injection at UPLC.

### Materials and solvents

Qseltamivir phosphate (OSTP), dexamethasone (DEX), daclatasvir dihydrochloride (DAC), and remdesivir (REM) standard powders were obtained from NODCAR, Cairo, Egypt. Their purities were assessed against the reported methods^8,22,26,36^ and they were found to be 99.976% w/w, 99.307% w/w, 101.171% w/w, and 99.841% w/w, respectively.

OSELTAMIVIR hard gelatin capsules (Arab-caps, batch No. 589006 and 589,007, Alexandria, Egypt) were purchased from a local pharmacy. Each hard capsule contains 98.50 mg of OSTP (equivalent to 75.00 mg of oseltamivir). DEXAZONE tablets were purchased from a local pharmacy (Kahira Pharm. & Chem. Ind. Co., batch No. 2110244, Cairo, Egypt). Each tablet contains 0.50 mg of DEX. JAVIDACLA film-coated tablets were kindly donated by Multicare Egypt for Pharmaceutical Industries, batch No. 160404 (Cairo, Egypt). Each film-coated tablet contains 65.92 mg of DAC, equivalent to 60.00 mg of DAC. Aliquots (100 µL) of ten REMDESIVIR vials (Eva Pharma, batch No. 2105418, Giza, Egypt) were separately obtained from Mahalla General Hospital, Egypt. Each vial of 20 mL of concentrated solution contains 100.00 mg of REM.

Methanol HPLC grade (Fisher Scientific, Germany) and ammonium acetate (AA) (Oxford laboratory reagent, India, min 96%) were purchased from CORNELL LAB—Fine Chemicals & LAB Equipment, Cairo, Egypt.

### Standard solutions

A stock solution containing a quaternary mixture of OSTP, DEX, DAC, and REM (1000 µg mL^−1^ for each drug) was prepared in a 10-mL volumetric flask using methanol as a solvent. The stock quaternary solution was further diluted with methanol to obtain a working solution of 500 µg mL^−1^ for each drug.

### General recommended procedures

#### Construction of calibration curves

Standard quaternary solutions with concentrations ranging from 10 to 30 µg mL^−1^ for each drug were made by transferring different aliquots of the working quaternary solution (500 µg mL^−1^ for each drug) into a series of 10-mL volumetric flasks, which were then completed to the mark with 30.0% (v/v) methanol. All prepared quaternary solutions were kept in the freezer (− 15 °C to − 28 °C) and shielded from light.

Volumes of 10 µL (in triplicate) were injected under optimal chromatographic conditions after filtering each prepared quaternary solution using a 0.22 mm membrane filter. To create the calibration curves, the peak areas were plotted against the final concentrations. The corresponding regression equations were then derived.

#### Stability of standard solutions

The UPLC vials containing laboratory-prepared standard quaternary solutions were frozen at (− 15 °C to − 28 °C). Each vial was analyzed by the proposed method at time intervals over eight days. For each drug, calibration curves were obtained every day. Percentages $(\frac{\mathrm{Slope \, at \, day \, x }(\mathrm{Sx})}{\mathrm{Slope \, at \, day \, 0 } (\mathrm{S}0) }\times 100)$ were plotted against the relevant days. The new regression equations predicted the appropriate period per day for storing standard and sample solutions in the freezer.

#### Commercial dosage forms

##### Estimation of average content

Five OSELTAMIVIR hard gelatin capsules were weighed (W_total_), then evacuated, and the evacuated powder was homogeneously mixed. The empty hard gelatin capsules were washed using diethyl ether and then dried in the air before being weighed (W_cap_). Then, 25 mL of methanol were used to transfer an accurately weighed amount of evacuated powder proportional to the estimated average weight ((W_total_ − W_cap_)/5) into a 100-mL volumetric flask. Another five JAVIDACLA film-coated tablets were weighed and then finely powdered. A carefully weighed amount equal to the average weight of five JAVIDACLA film-coated tablets was placed into the same flask with another 25 mL of methanol and sonicated for 15 min. After sonication, the flask was allowed to cool for 5 min before being filled to the mark with methanol (solution A).

Five DEXAZONE tablets were finely powdered and transferred quantitatively into a 10-mL volumetric flask using 5 mL of methanol. The flask was sonicated for 15 min and then kept at room temperature for 5 min before being filled with methanol to the mark (solution B). To reach the average content of the five DEXAZONE tablets, solution B was diluted to its fifth strength. 2 mL of solution B were transferred into a 10-mL volumetric flask, which was then filled with methanol to the mark (solution C).

To estimate remdesivir's average content, 25 µL of REMDESIVIR was transferred into an Eppendorf tube from each of the five vials and then vortexed for 30 s (solution D).

Finally, aliquots of 250 µL, 500 µL, and 25 µL were transferred from solutions A, C, and D, respectively, into 10-mL volumetric flasks and completed with 30.0% (v/v) methanol (solution E). Later, solution E was diluted by transferring an aliquot of 100 µL into a 10-mL volumetric flask, which was then filled to the mark with 30.0% (v/v) methanol (solution F).

The suggested method was used to analyze the final quaternary mixes (solutions E and F) after filtration through a 0.22-mm membrane filter. The concentrations of the four drugs were determined using the corresponding regression equations.

##### Evaluation of uniformity of dosage units

The above procedure under the "Estimation of average content" section was adopted for each intact dosage form (ten units of each preparation were used).

Ten OSELTAMIVIR hard gelatin capsules were collectively (W_total_) and individually (Wn) weighed. The empty hard gelatin capsules were separately washed with diethyl ether, and the washings were transferred into ten test tubes. Both the empty capsules and the ten test tubes were dried by air, and then the empty capsules were weighed (W_cap_) and separately weighed (Wn_cap_). Then one residue from a dried test tube was transferred into a 100-mL volumetric flask with its corresponding evacuated powder using 50 mL of methanol. One JAVIDACLA film-coated tablet was inserted in this flask, and the flask was sonicated for 15 min. After sonication, the flask was allowed to cool for 5 min before being filled to the mark with methanol (solution G).

One DEXAZONE tablet was sonicated until totally disintegrated in an Eppendorf tube containing 0.5 mL of methanol. Using 5 mL of methanol, the product was quantitatively transferred into a 10-mL volumetric flask. The flask was sonicated for 15 min and then kept at room temperature for 5 min before being filled with methanol to the mark (solution H).

Finally, aliquots of 250 µL, 500 µL, and 25 µL were transferred from solutions G, H, and one REMDESIVIR vial, respectively, into a 10-mL volumetric flask and marked with 30.0% (v/v) methanol (solution I). Later, solution I was diluted by transferring an aliquot of 100 µL into a 10-mL volumetric flask, which was then filled to the mark with 30.0% (v/v) methanol (solution J). This procedure was repeated for the rest of the nine dosage units.

The resultant quaternary mixtures (solutions I and J) were filtered through a 0.22-mm membrane filter and analyzed by the suggested method. The concentrations of the four drugs were determined using the regression equations that corresponded to them.

##### Procedure of the classic standard addition method

Two procedures were used. The first procedure was applied to the low linearity ranges of OSTP, DAC, and REM. This was accomplished by transferring various aliquots (0, 10, 100, 250, and 500 µL) of the standard quaternary solution (250 ng mL^−1^) into a series of 10-mL volumetric flasks holding 100 µL of solution E and then filling them to the mark with 30.0% (v/v) methanol.

The second procedure was used for OSTP, DAC, and REM at high linearity ranges and also for DEX. This was accomplished by transferring various aliquots (0, 10, 100, 250, and 500 µL) of the standard solutions (500 µg mL^−1^) into a series of 10-mL volumetric flasks holding aliquots of 250 µL, 500 µL, and 25 µL from solutions A, C, and D, respectively, and then completing them with 30.0% (v/v) methanol.

The resulting solutions were filtered through a 0.22-mm membrane filter and analyzed using the proposed method.

The final standard concentrations (w/v) were estimated using “Eq. (1)” and plotted against the corresponding responses (AUC).

1 $$\mathrm{The \, final \, standard \, concentration }(\mathrm{w}/\mathrm{v}) =\frac{\mathrm{Standard \, solution \, concentration }(\mathrm{w}/\mathrm{v})}{\mathrm{Flask \, volune}(\mathrm{mL})}\times \mathrm{corresponding \, aliquot }(\mathrm{mL})$$

From the corresponding regression equations, the average content concentrations of the four drugs were calculated [Eq. (2)]:

2 $$\mathrm{Average \, content \, concentration},\mathrm{X}(\mathrm{w}/\mathrm{v}) =\frac{-(0-intercept)}{slope}$$

##### Procedure of the innovative instrumental standard addition method

This was performed using the UPLC instrument program. Two vials, one containing a standard quaternary solution (250 ng mL^−1^ for each drug) and the other containing solution E, were utilized. The UPLC was programmed to mix various aliquots (0, 2, 3, 4, 5, and 6 µL) of the first vial with 0.1 µL of the second (instead of taking 10 µL from a single vial as in the classic standard addition method), then analyze them using the proposed method. The final standard amounts (ng) were estimated by “Eq. (3)” and plotted against the corresponding responses (AUC).

3 $$\mathrm{The \, final \, standard \, amounts }(\mathrm{ng}) =\frac{250 ({ng mL}^{-1})}{1000}\times \mathrm{corresponding \, aliquot }({\mu L})$$

Using the corresponding regression equations, the average content concentrations of the four preparations were calculated using “Eq. (2)”.

#### Experimental design (DOE)

DOE refers to the process of making systematic plans to extract maximum data with minimum experimentation. From these extracted data, meaningful conclusions are drawn using statistical models^30^. The full factorial design (2^3^ FFD), response optimizer, optimization plot, and estimated coefficients of the independent factors (data in uncoded units) were studied in this work.

In the full factorial design (FFD), three factors with two levels were chosen. As a result, eight designed experiments (2^3^ total) were conducted. In the response optimizer, the lower, target, and higher values for dependent responses as well as their importance values were determined. The importance value has a range of 0.1 to ten. Because all responses are equally significant, the default value of one was applied to each.

The response optimizer calculated the optimal setting of the input variables as well as the values of individual desirability (d) and composite desirability (D). The optimal UPLC conditions with the highest composite desirability (D) value were determined. Individual desirability (d) measures how well the conditions optimize a single response, whereas composite desirability (D) measures how well the conditions optimize all responses in the set. Desirability is measured on a scale of zero to one.

The optimization plot showed the effect of each independent factor (columns) on the dependent responses (rows). The vertical red lines on the graph represent the optimum settings of the independent factors. The red numbers displayed at the top of the columns showed the optimum values of the independent factors. The horizontal dashed blue lines and numbers (y) represented the responses to the optimum settings (Table S1).

Furthermore, factorial design analysis provides estimated coefficients of the independent factors (data in uncoded units), from which mathematical equations [Eqs. (S1)–(S12)] for chromatographic responses can be created. These equations can predict the proposed method's robustness^31^ and system suitability parameters.

## Results and discussion

### Method development and optimization

After many experimental trials, well-defined symmetrical peaks were achieved. These experiments can be summarized as follows:

#### Method development

##### Selection of column and guard column

The most common UPLC separations use the 1.7-µm UPLC Ethylene (-CH_2_-CH_2_-) Bridged Hybrid (BEH) C_18_ columns as the first choice. BEH groups have three functions. The first function is due to the ability of the surface BEH groups to conceal the surface free silanol groups, which results in improving the tailing factor and, thus, the peak shape. The second function is due to the cross-linking BEH groups, which improve the column's chemical and mechanical stability. The third function is due to the internal hydrophobic BEH groups that have the best pH stability in the range of pH 1.0 to 12.0. These trifunctionally bonded alkyl columns can use the power of pH to influence the retention, selectivity, and sensitivity of ionizable molecules. They enable a robust separation technology for method development^40^.

Guard columns are intended for routine usage in UPLC systems. For many reasons, they preserve and extend the life of UPLC columns while providing minimum chromatographic effects. They are designed for routine use at pressures up to 15,000 psi [1000 bar]. Their ultra-low volume design (2.1-mm-ID × 5-mm-length) efficiently preserves UPLC column performance. Furthermore, during the manufacturing of guard columns, the same UPLC column stationary phase and column frits are used, resulting in an improvement in the overall separation efficiency and resolution. Finally, the guard columns are connected directly to the UPLC column's inlet, so extra-column volumes are minimized, and mobile-phase leaks caused by additional connections are almost avoided^41^.

##### Selection of the mobile phase

The retention time and peak tailing are greatly affected by the pH of the mobile phase. The eco-friendly biodegradable methanol was intended to be utilized as the organic mobile phase (pH 7.0–8.3^42^). On the other hand, the aqueous mobile phase was intended to be water (pH 7.0), 0.1% (v/v) acetic acid (pH 4.3), or ammonium acetate solution (pH 7.0^43^). Water is widely available, inexpensive, and eco-friendly. Ammonium acetate solution is widely available, chemically stable, non-toxic, and inexpensive. It is totally ionized, almost neutral in water, and an effective buffering medium. It is extremely soluble in methanol and acetonitrile and serves as an effective masking agent for residual silanol groups on the chromatographic media, resulting in greatly enhanced separations. It can speed up proton equilibrium rates, which is essential for the chromatography of ionic compounds. It is also relatively volatile and easy to remove after preparative separation or when used with a mass spectrometer. It is compatible with all frequently used liquid chromatographic detectors^44^.

From the previous, it was predicted that the overall pH range of methanol with the selected aqueous mobile phases was 4.8 ± 0.5 (in the case of 0.1% (v/v) acetic acid) or 7.5 ± 0.5 (in the case of water and ammonium acetate).

##### Selection of the optimum wavelength

The wavelength of the analysis should be at least 20 nm above the solvent's UV cutoff^34,45^. Both methanol and ammonium acetate have UV cutoffs at 205 nm, while acetic acid solution has a UV cutoff at 210 nm. As a result, the PDA-detector should theoretically be set above 230 nm to reduce baseline noise during the baseline stabilization process^46,47^. It was found that the absorbance at 240 nm of the degassed methanol, 0.1% (v/v) acetic acid, and 10.0000 mM ammonium acetate is 0.05, 0.01, and less than 0.01, respectively^48^. As a result, during optimization, data was taken at 239 or 240 nm from the PDA scan (Fig. S2 and Table S2).

##### Prediction of the drugs’ order of separation

The compounds' log P (log Kow) and pka values influence their separation order^49^. OSTP, DEX, or its phosphate salt (DEXP), REM, and DAC were the drugs under investigation. Theoretically, according to log P (Table S3), these drugs would elute in the following order: OSTP, DEX/DEXP, REM, and then DAC. However, based on their PKa values in addition to their log P, the sequence would be DEXP, OSTP, DEX, DAC, and finally REM. This is due to the fact that ionizable compounds have significantly shorter retention times than unionizable compounds in reversed-phase high-performance liquid chromatography^49^.

It was observed that the log P values of DEXP, DEX, and OSTP are close, as are the log P values of DAC and REM. As a result, the ionization of these drugs was the primary factor influencing their elution sequence. The drugs under investigation had both acidic and basic functional groups. Table S3 shows that at pH 4.8 ± 0.5 and 7.5 ± 0.5, DEXP was more ionized than OSTP, while OSTP was more ionized than DEX. As a result, DEXP would elute before OSTP, and OSTP would elute before DEX. The capacity factor (*k*′) of DEXP created during optimization would determine which of DEXP and DEX should be used. DAC acidic and basic groups, on the other hand, would be ionized, and REM molecules would be totally unionized. As a result, it was predicted that DAC would be eluted before REM.

##### Difficulties anticipated with UPLC analysis

Two difficulties were anticipated with UPLC analysis. The first was due to the high degree of ionization of each of DEXP, DAC, and OSTP. It was hypothesized that when using water as an aqueous mobile phase, they would produce tailed peaks. This is due to interactions between ionized DEXP, DAC, and OSTP and free silanol groups. So, it was necessary to use a low pH (0.1% v/v) acetic acid solution or an ammonium acetate solution to hide free silanol groups in the stationary phase and hence decrease tailing (T).

The second difficulty was peak overlap between DEXP and OSTP, between OSTP and DEX, and between DAC and REM due to their close log P values and use of methanol as an organic mobile phase. This problem was solved by gradually decreasing the flow rate while increasing the methanol percentage.

##### UPLC-PDA method development

Over the course of 13 days, 23 different conditions were used to develop the method (Table S2). Method development was first carried out on REM to calculate the total run time, then on OSTP and DEXP to calculate the new method's capacity factor (*k*').

At room temperature, the peak of REM (100 µg mL^−1^ dissolved in 60.0% (v/v) methanol) was detected at 37.500 min (min) with a flow rate of 0.200 mL min^−1^ and a mobile phase of methanol and water (50:50). Under identical conditions, the peak of a freshly prepared OSTP solution (100 µg mL^−1^ dissolved in 50.0% (v/v) methanol) was detected at 15.197 min. So, the resulting capacity factors (*k*') of REM and OSTP were very high (Table S2, Method 1).

As a result, it was suggested to increase the methanol percentage in the mobile phase to 60.0% (v/v) (Table S2, Method 2). Hence, the REM peak was detected at 7.600 min, while the OSTP peak was detected at 6.260 min. The REM peak had a good number of theoretical plates (N), while the OSTP peak was very broad [high width (W)] with a high T and a low N. So, the methanol percentage in the mobile phase was increased again to decrease W and T and increase N (Table S2, Methods 3 and 4). Unfortunately, methods 3 (methanol:water = 70:30) and 4 (methanol:water = 80:20) showed slight improvements in W, T, and N of the OSTP peak.

So, 0.1% (v/v) acetic acid (pH 4.2) was used with methanol instead of water to mask free silanol groups and hence decrease T and W of the OSTP peak and hence increase its N (Table S2, Methods 5, 6, and 7). According to Methods 5, 6, and 7 (Table S2), OSTP peak’s T was significantly reduced, but its W remained high, and thus its N was low. As a result, it was recommended to use ammonium acetate solution with methanol rather than 0.1% (v/v) acetic acid (Table S2, Methods 8, 9, and 10). Ammonium acetate can reduce retention times and improve peak shape not only by deactivating the free silanols of the stationary phase but also by forming ion pairs with the analyte^50^.

During Methods 8, 9, and 10 (Table S2), the temperature of the column chamber was kept constant at 25 °C while 10.0000 mM ammonium acetate (pH 7.0 ± 0.2) with different ratios of methanol at a flow rate of 0.150 mL min^−1^ was tested. They exhibited promising system suitability parameters for OSTP. Therefore, under the conditions of Method 10 (Table S2), REM and DEXP were tested separately as well as in their ternary mixture (DEXP, OSTP, and REM). The retention times of DEXP, OSTP, and REM were found to be 2.795, 3.681, and 10.471 min. OSTP and REM showed good system suitability parameters, while DEXP showed a high T. This tailing was suggested to be solved by increasing the concentration of ammonium acetate to 20.0000 mM (Table S2, Method 11) and subsequently increasing the flow rate (Table S2, Method 12), but T remained high. The flow rate was gradually decreased with an increase in the methanol percentage in the mobile phase (Table S2, Methods 13 to 18). Unfortunately, T of DEXP remained high (> 2). Besides, its *k*′ was very low (0.2).

The following action was to change DEXP to DEX. According to Table S3, DEX would be completely unionized at pH 7.5 ± 0.5, resulting in an acceptable tailing value for the DEX peak. Three DEX solutions (4.0 µg mL^−1^ each) in three different solvents were separately prepared from a methanolic stock solution of DEX, and then they were analyzed by Method 18 (Table S2). The resulted DEX peaks had good system suitability compared to DEXP. Additionally, their area under the curve responses (AUC) were enhanced as the methanol percentage in the diluting solvent increased, while noisy baselines were produced in the case of diluting by mobile phase.

A ternary mixture of OSTP, DEX, and REM in 10.0% (v/v) methanol was then tested. The system suitability parameters of their resulted peaks were good. Therefore, DAC was evaluated both alone and then in combination with DEX, OSTP, and REM by Method 18 (Table S2). Their T, N, *k*', and resolution (R_S_) values were found to be promising.

Finally, Methods 18–23 (Table S2) were tested in order to identify the factorial design factors and their levels.

##### UPLC-PDA analysis of factorial designs

During the UPLC analysis of factorial designs, the quaternary mixtures were diluted with 30.0% (v/v) methanol. This was because samples diluted with 30.0% (v/v) methanol had higher responses (higher AUC) than ones diluted with water (Table S2; Trials 30 and 31; and Trials 45 to 48). This was due to the drugs' poor water solubility (Table S3), which led to the adsorption of the tested drugs in the syringe filter during filtration.

In addition, five wave lengths were evaluated during the UPLC analysis of the factorial designs based on PDA scanning (200–400 nm) of the analyzed drugs (Fig. S2) to determine the most suitable wavelength^31^. These wavelengths (λ) were 239 nm (λ_max_ of DEX), 245 nm (λ_max_ of REM), 315 nm (λ_max_ of DAC), 274 nm (DEX, DAC, and REM only), and 340 nm (OSTP, DEX, and DAC only). It was found that 239 nm was the most suitable one with satisfactory responses for the four drugs (Fig. S3). As a result, the dependent responses generated from chromatograms at 239 nm were optimized (Table S5).

##### Novel Determination of the stability of standard solutions

Stabilities per day were determined for each drug according to the procedure mentioned under the "Stability of standard solutions" section. All stock and standard solutions were frozen for about three weeks for OSTP and DEX and one week for each of DAC and REM.

##### Novel assessment of excipients’ interference

The interference of excipients was assessed using the classical standard addition method and the innovative instrumental standard addition method. The instrumental standard addition method outperformed the traditional method in that it was more accurate and sensitive because it was more dependent on the instrument (mixing procedure); it was time-saving and had fewer human errors because only one sample and one standard were needed to be prepared; it was economical because it required only a small amount of solvents; and thus, it was more eco-friendly. The only disadvantage was that the UPLC had to be calibrated.

##### Method optimization by full factorial design

The chromatographic performance was found to be affected by three independent factors: methanol percentage (v/v) [meth %; A; ranged from 73.0 to 77.0% (v/v)], ammonium acetate concentration mM (AA conc.; B; ranged from 5.0000 to 20.0000 mM), and flow rate mL min^−1^ (FR; C; ranged from 0.030 to 0.070 mL min^−1^) (Table S2; methods 18 to 23 and Table S4). According to Table S4, the most dependent responses impacted by the independent factors were *k*′ _(OSTP)_, T_1 (OSTP)_, and R_S3 (REM)_.

The *k*′ _(OSTP)_, T_1 (OSTP)_, and R_S3(REM)_ results for all eight designed experiments were within the (0.48–0.91), (1.30–2.30), and (1.39–2.83) ranges. However, the acceptable limit for *k*' is greater than 0.5^51^, T is from 0.8 to 2, and R_S_ is greater than 1.5^52^. As a result, these dependent responses were fed into the response optimizer program. The lower and target values for *k*′ _(OSTP)_, T_1 (OSTP)_, and R_S3 (REM)_ (Table S1) were entered into the response optimizer program, and the optimum conditions for the input values and desirability values were obtained^31^.

The final optimum condition, according to the response optimizer and optimization plots (Table S1)^31^, was 75.7% (v/v) methanol with 24.3% (v/v) AA (8.1818 mM) at a flow rate of 0.048 mL min^−1^ and an injection volume of 10 mL (Table 1).

**Table 1:** Optimum chromatographic conditions for the RP-UPLC-PDA separation of oseltamivir phosphate/dexamethasone/daclatasivir dihydrochloride/remdesivir mixture.

Column	UPLC column BEH C_18_ 1.7 µm (2.1 × 100 mm) connected with UPLC guard-column BEH 1.7 µm (2.1 × 5 mm)
Mobile phase	Methanol : Ammonium acetate (8.1818 mM) = 75.7: 24.3 (v/v)
Full run time	9.5 min
Flow rate	0.048 mL min^−1^
Temperature	Ambient (25 °C) at column oven and autoinjector part
Injection volume	10 µL
PDA detector	239 nm

### Factors affecting *k*′_(OSTP)_, T_1(OSTP)_, and R_S3(REM)_ according to 2^3^-FFD

Three independent factors were shown to have an effect on *k*′_(OSTP)_, T_1(OSTP)_, and R_S3(REM)_, which were methanol percentage (A), ammonium acetate concentration in mM (B), and flow rate in mL min^−1^ (C).

#### Factors affecting *k*′_(OSTP)_

**Figure 1:**
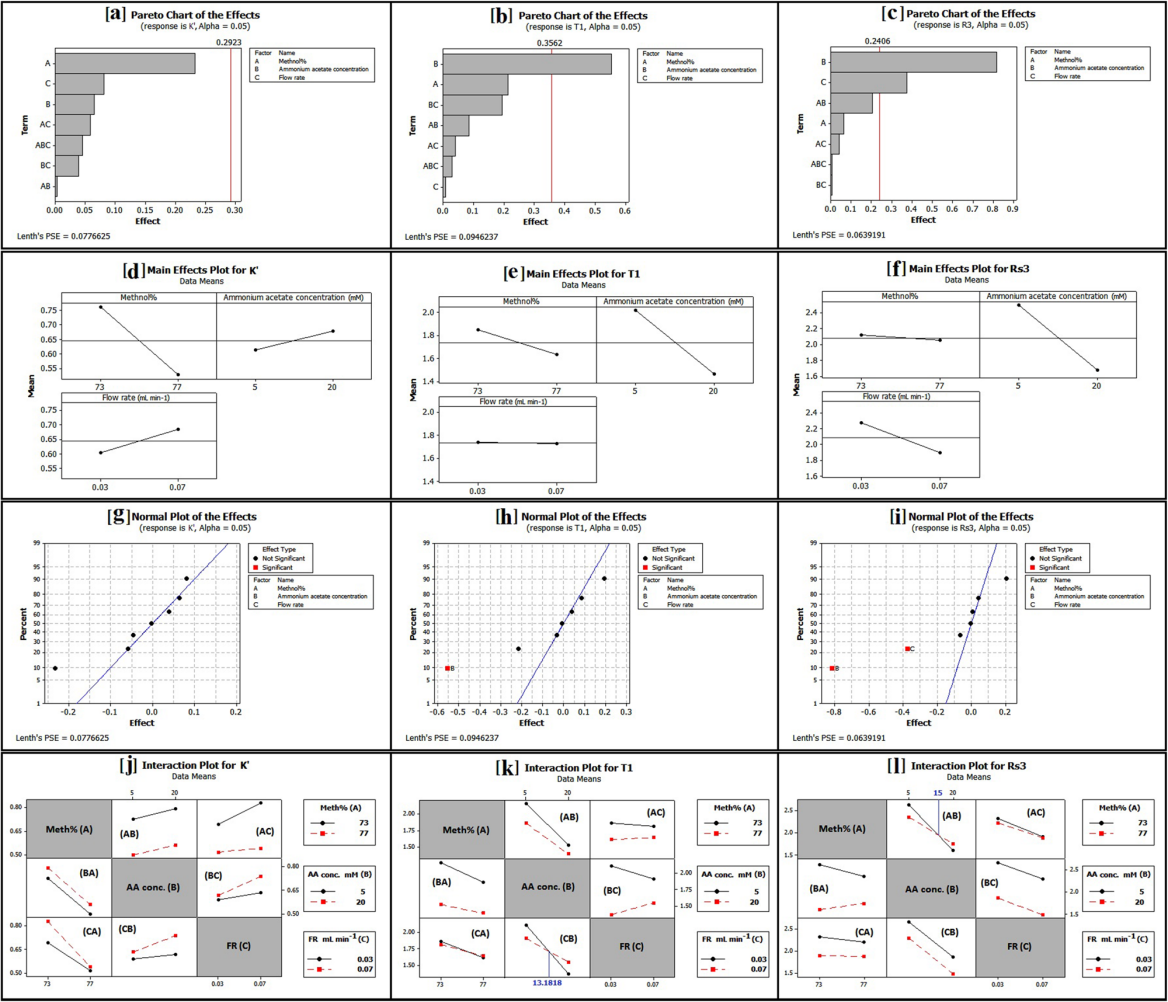
2^3^ FFD pareto charts (**a**–**c**) and normal plots (**g**–**i**) of the effects on the chromatographic responses at alpha = 0.05, and main effect plots (**d**–**f**) and interaction plots (**j**–**l**) for chromatographic responses by data means type**.**

The Pareto chart of standardized effects (Fig. 1a) demonstrated that methanol percentage (A) had the biggest influence on *k'*_(OSTP)_ since it was the longest, but it was not statistically significant at a 95% confidence level. This was supported by its sharp slope in the main effects plot (Fig. 1d) and the highest absolute values of estimated effects (Table S5). Based on the estimated effects (Table S5) and the normal plot of the effects (Fig. 1g), the effect of the methanol percentage (A) was negative.

This meant that as the percentage of methanol (A) increased, *k'*_(OSTP)_ decreased because OSTP interacted more effectively with methanol than the reversed C_18_ stationary phase, which was consistent with the solvophobic theory^53,54^. Solvophobic theory describes the hydrophobic interaction among analytes, the stationary phase, and the solvent^55^.

According to the interaction plot (Fig. 1j), a similar negative effect was seen when methanol percentage interacted with ammonium acetate concentration (BA interaction) or flow rate (CA interaction) at their high and low levels. At the BA interaction, the high and low concentrations of ammonium acetate could hardly interact since they were on parallel lines, and the opposite was the case for the CA interaction. Furthermore, *k'*_(OSTP)_ values were larger when methanol percentage interacted with 20.0000 mM ammonium acetate or a 0.070 mL min^−1^ flow rate based on BA and CA interactions, respectively.

According to the Pareto chart (Fig. 1a) and the interaction plot (Fig. 1j), the interactions with the greatest and least effect on *k'*_(OSTP)_ were AC (flow rate with 73.0 or 77.0% (v/v) methanol) and AB (ammonium acetate concentration with 73.0 or 77.0% (v/v) methanol), respectively. Moreover, flow rate and ammonium acetate concentration, when interacting with methanol percentage at AC and AB interactions, both had a positive influence on *k'*_(OSTP)_. Additionally, at 73.0% (v/v) methanol, they produced greater *k'*_(OSTP)_ values than at 77.0% (v/v) methanol.

Finally, the interaction plot (Fig. 1j) revealed that the maximum value of *k'*_(OSTP)_ was at 73% (v/v) methanol (from AB, AC, BA, and CA interactions), 20.0000 mM ammonium acetate (from AB, BC, BA, and CB interactions), and a flow rate of 0.070 mL min^−1^ (from AC, BC, CA, and CB interactions). However, to maintain the optimum conditions, 75.7% (v/v) methanol, 8.1818 mM ammonium acetate, and a flow rate of 0.048 mL min^−1^ were used.

#### Factors affecting T_1(OSTP)_

The Pareto chart of standardized effects (Fig. 1b) demonstrated that ammonium acetate concentration (B) had the biggest influence on T_1(OSTP)_ since it was the longest, and it was statistically significant at a 95% confidence level. This was supported by its sharp slope in the main effects plot (Fig. 1e) and the highest absolute value of estimated effects (Table S5). Based on the estimated effects (Table S5) and the normal plot of the effects (Fig. 1h), the effect of ammonium acetate concentration (B) was negative. This meant that when the concentration of ammonium acetate (B) increased, the T_1(OSTP)_ decreased. This was because the addition of ammonium acetate rendered the stationary phase's free silanols inactive^44,50^.

According to the interaction plot (Fig. 1k), a similar negative influence was seen when ammonium acetate concentration interacted with methanol percentage (AB interaction) or flow rate (CB interaction) at their high and low levels. Furthermore, T_1(OSTP)_ values were smaller when ammonium acetate concentration interacted with 77.0% (v/v) methanol based on the AB interaction. The interaction of ammonium acetate concentration with flow rate (CB interaction), on the other hand, was divided into two phases. T_1(OSTP)_ values were lower when ammonium acetate concentrations ranging from 5.0000 to 13.1818 mM interacted with a 0.070 mL min^−1^ flow rate, as well as when ammonium acetate concentrations ranging from 13.1818 to 20.0000 mM interacted with a 0.030 mL min^−1^ flow rate.

According to the Pareto chart (Fig. 1b) and interaction plot (Fig. 1k), the interactions with the greatest and least effects on *k'*_(OSTP)_ were BC (flow rate with 5.0000 or 20.0000 mM ammonium acetate) and AC (flow rate with 73 or 77.0% (v/v) methanol), respectively. Furthermore, ammonium acetate concentration and methanol percentage both affected T_1 (OSTP)_ positively at their high levels and negatively at their low levels when they interacted with flow rate at BC and AC interactions.

Finally, the interaction plot (Fig. 1k) revealed that the minimum value of T_1(OSTP)_ was at 77.0% (v/v) methanol (from AB, AC, BA, and CA interactions), 20.0000 mM ammonium acetate (from AB, BC, BA, and CB interactions), and a flow rate of 0.030 mL min^−1^ (from AC, BC, CA, and CB interactions). However, to maintain the optimum conditions, 75.7% (v/v) methanol, 8.1818 mM ammonium acetate, and a flow rate of 0.048 mL min^−1^ were used.

#### Factors affecting Rs_3 (REM)_

The Pareto chart of standardized effects (Fig. 1c) demonstrated that ammonium acetate concentration (B) had the biggest influence on Rs_3 (REM)_ since it was the longest, and it was statistically significant at a 95% confidence level. This was supported by its sharp slope in the main effects plot (Fig. 1f) and the highest absolute value of estimated effects (Table S5). Additionally, flow rate (C) had the second-largest statistically significant impact on Rs_3 (REM)_ (Fig. 1c).

Based on the estimated effects (Table S5) and the normal plot of the effects (Fig. 1i), the effects of ammonium acetate concentration (B) and flow rate (C) were negative. This means that when ammonium acetate concentration (B) and flow rate (C) increased, R_S3 (REM)_ decreased.

Ammonium acetate acted as an ion pairing additive^44^. The ionization of DAC molecules into DAC^+^ cations occurred at pH 7.5 ± 0.5 (Table S3). As a result, less polar ion pairs with a delayed retention time were produced when the acetate anions interacted with the DAC^+^ cations. The retention time of the unionized REM, on the other hand, was reduced because ammonium^+^ cations masked free silanol groups in the stationary phase, preventing hydrogen bonds from forming between the free silanols and the nitrogen atoms of REM^50,56^. As a result, increasing the concentration of ammonium acetate decreased the resolution between DAC and REM.

According to the interaction plot (Fig. 1l), a similar negative effect was seen when ammonium acetate concentration interacted with methanol percentage (AB interaction) or flow rate (CB interaction) at their high and low levels. At the CB interaction, the high and low flow rates could hardly interact since they were parallel lines. The interaction of ammonium acetate concentration with methanol percentage (AB interaction), on the other hand, was divided into two phases. Rs_3 (REM)_ values were higher when ammonium acetate concentrations ranging from 5.0000 to 15.0000 mM interacted with 73.0% (v/v) methanol, as well as when ammonium acetate concentrations ranging from 15.0000 to 20.0000 mM interacted with 77.0% (v/v) methanol.

According to the Pareto chart (Fig. 1c) and interaction plot (Fig. 1l), the interactions with the greatest and least effects on Rs_3 (REM)_ were AB (ammonium acetate concentration with 73.0 or 77.0% (v/v) methanol) and BC (flow rate with 5.0000 or 20.0000 mM ammonium acetate), respectively. Moreover, ammonium acetate concentration and methanol percentage, when interacting with flow rate at BC and AC interactions, both had a negative effect on Rs_3 (REM)_. Furthermore, at their low levels, they yielded lower Rs_3 (REM)_ values than at their high ones.

Finally, the interaction plot (Fig. 1l) revealed that the maximum value of Rs_3 (REM)_was at 73.0% (v/v) methanol (from AB, AC, BA, and CA interactions), 5.0000 mM ammonium acetate (from AB, BC, BA, and CB interactions), and a flow rate of 0.030 mL min^−1^ (from AC, BC, CA, and CB interactions). However, to maintain the optimum conditions, 75.7% (v/v) methanol, 8.1818 mM ammonium acetate, and a flow rate of 0.048 mL min^−1^ were used.

### Method characteristics

The reversed phase UPLC-PDA technique was developed for simultaneous determination OST, DEX, DAC, and REM. For the selection of the factors affecting the method, a mixture comprising 20 µg mL^−1^ of each drug was prepared. The mixture's 2^3^ full factorial designs were applied using 30.0% (v/v) methanol as a blank and saved in the computer. The dependent factors at 239 nm were determined using the chromatograms of the combination (Table S4).

### Method validation

The proposed method was validated in accordance with the ICH guidelines^57^. The following validation parameters were evaluated:

#### Linearity

A PDA-detector analysis of six concentrations was used to assess the method's linearity for each drug (Table 2). The suggested method's calibration curves were achieved within the stated linearity limits for each drug, as shown in Table S6. Table 2 and Fig. S4 summarize the quantitative statistical criteria for the determination of OSTP, DEX, DAC, and REM. The high coefficients of determination (R^2^ > 0.9999) suggested that the calibration curves were linear.

**Table 2:** Statistical parameters of calibration curves of oseltamivir phosphate/dexamethasone/daclatasvir dihydrochloride/remdesivir using the developed method.

Parameter	Oseltamivir phosphate		Dexamethasone	Daclatasvir dihydrochlorde		Remdesivir	
	Low linearity range	High linearity range	Linearity range	Low linearity range	High linearity range	Low linearity range	High linearity range
Linearity range	10–500 (ng mL^−1^)	0.5–30 (µg mL^−1^)	50–5000 (ng mL^−1^)	25–1000 (ng mL^−1^)	0.5–25 (µg mL^−1^)	10–500 (ng mL^−1^)	0.5–30 (µg mL^−1^)
Intercept (a)	− 203.879	− 4720.117	4362.943	850.238	1455.288	4574.515	− 119,660.598
Slope (b)	80.859	89,012.348	326.194	109.500	110,422.852	673.587	634,283.236
Coefficient of determination (R2)	0.999966	0.999997	0.999995	0.999990	0.999995	0.999975	0.999961
SD of intercept (Sa)	55.290	1263.135	831.417	81.157	1464.841	392.926	28,719.258
SD of slope (Sb)	0.235	73.413	0.358	0.172	125.783	1.672	1988.734
SD of residuals (Sy/x)	99.193	2077.570	1563.466	143.894	2678.239	704.933	49,554.018
LOD^a^	2.256^b^, 2.572^c^, 1.923^d^	0.047^b^, 0.046^c^	8.411^b^, 0.840^c^, 0.543^d^	2.446^b^, 2.004^c^, 2.340^d^	0.044^b^, 0.045^c^	1.925^b^, 1.097^c^, 0.478^d^	0.149^b^, 0.106^c^
LOQ^a^	6.838^e^, 7.793^f^, 6.412^g^	0.142^e^, 0.138^f^	25.488^e^, 2.546^f^, 1.809^g^	7.412^e^, 6.073^f^, 7.802^g^	0.133^e^, 0.137^f^	5.833^e^, 3.326^f^, 1.593^g^	0.453^e^, 0.323^f^
Mean recovery ± SD, %	99.801 ± 1.060	99.595 ± 0.935	99.828 ± 0.597	100.254 ± 1.014	99.680 ± 0.648	99.833 ± 1.141	100.322 ± 0.976
RSD, %^h^	1.062	0.939	0.598	1.012	0.650	1.143	0.973
Error, %^i^	0.433	0.382	0.244	0.414	0.290	0.466	0.398

^a^Its units are ng mL^−1^ for DEX and low linearity OSTP, DAC, and REM ranges, and µg mL^−1^ for high linearity OSTP, DAC, and REM ranges.

^b^Limit of detection based on the standard deviation of the y-intercept (σ) and the slope of a regression line (S) of the linear calibration curve; LOD = 3.3 σ/S.

^c^Limit of detection based on the standard deviation of the y-intercept (σ) and the slope of a regression line (S) of a specific calibration curve in the range of LOD of the linear calibration curve; LOD = 3.3 σ/S.

^d^Limit of detection based on signal-to-noise ratios (S/N) method.

^e^Limit of quantitation based on the standard deviation of the y-intercept (σ) and the slope of a regression line (S) of the linear calibration curve; LOQ = 10 σ/S.

^f^Limit of quantitation based on the standard deviation of the y-intercept (σ) and the slope of a regression line (S) of a specific calibration curve in the range of LOD of the linear calibration curve; LOQ = 10 σ/S.

^g^Limit of quantitation based on signal-to-noise ratios (S/N) method.

^h^Percentage relative standard deviation for six samples.

^i^Percentage relative error for six samples.

#### Limits of detection and quantitation

They were calculated using two methods, according to ICH recommendations (Q2 R1)^57^:

The first was the S/N ratio method, which used peak height data. In this method, a signal-to-noise ratio (S/N) of 3:1 was applied for the limits of detection (LOD) and 10:1 for the limits of quantitation (LOQ). The second method involved the use of the following “Eqs. (4) and (5)”:

4 $$\mathrm{LOD }=3.3\upsigma /\mathrm{S}$$

5 $$\mathrm{LOQ }=10\upsigma /\mathrm{S}$$

where the standard deviations of the y-intercept (σ) and the slope of a regression line (S) are either of the linear calibration curve or of a specific calibration curve in the range of LOD and LOQ.

**Figure 2:**
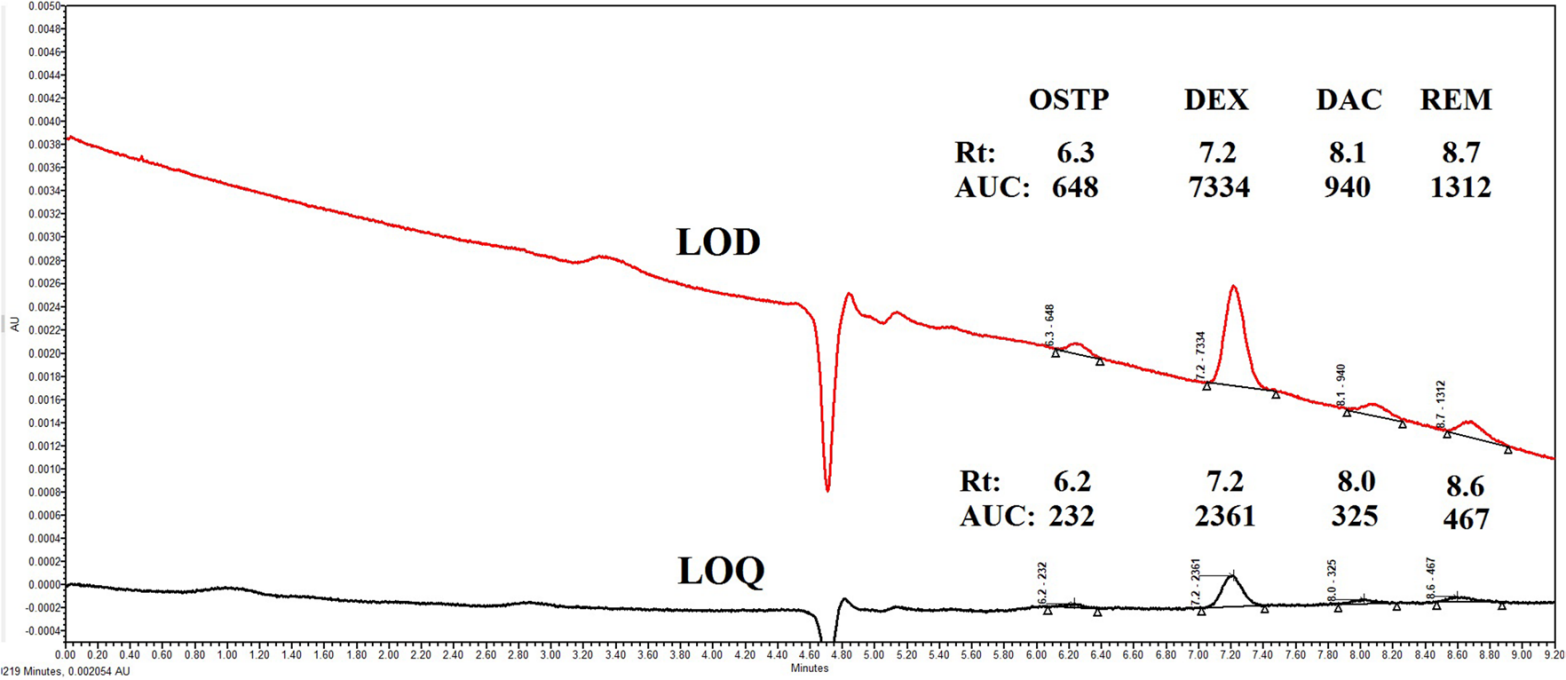
Chromatograms by the proposed method for: Limit of detection (LOD): ≈ 2 ng mL^−1^ OSTP, ≈ 8 ng mL^−1^ DEX, ≈ 2 ng mL^−1^ DAC, and ≈ 0.5 ng mL^−1^ REM. Limit of quantitation (LOQ): ≈ 6 ng mL^−1^ OSTP, ≈ 25 ng mL^−1^ DEX, ≈ 6 ng mL^−1^ DAC, and ≈ 1.6 ng mL^−1^ REM.

The LOD and LOQ values for the proposed method are shown in Table 2 and Fig. 2. The developed method has a sensitivity of 1.9 ng mL^−1^ for OSTP, 0.5 ng mL^−1^ for DEX, 2.0 ng mL^−1^ for DAC, and 0.5 ng mL^−1^ for REM, so the proposed method is suitable for their assay in human plasma. To achieve these low concentrations in plasma, solid-phase extraction (SPE), liquid–liquid extraction (LLE) with the salting-out technique, or liquid-phase microextraction (LPME)^58,59^ are excellent choices. They clean up the sample and preconcentrate the target analytes in very small volumes. This increases the concentration to a limit that can be detected by UV or PDA detectors. In addition, these techniques reduce the consumption of organic solvents, saving time, effort, and money.

#### Accuracy and precision

The suggested method's accuracy and precision (intra-day and inter-day) were determined using three separate laboratory-prepared quaternary mixtures covering low, middle, and high levels at each linearity range (three replicates) (Table S7). The obtained concentrations (conc. found, %) were compared to those obtained using the comparison methods^8,22,26,36^. The standard deviation (SD) and percentage relative standard deviation (% RSD) of the obtained findings were calculated. The RSD was found to be extremely small (less than 1.69% for OSTP, 1.22% for DEX, 1.80% for DAC, and 1.90% for REM), indicating that the proposed method had appropriate accuracy and precision (Table S7).

The findings obtained by the suggested and reported methods^8,22,26,36^ indicated no significant differences in statistical analysis (Student's t-test and Variance Ratio F-test) (Tables S6 and S8).

#### Selectivity and specificity

**Figure 3:**
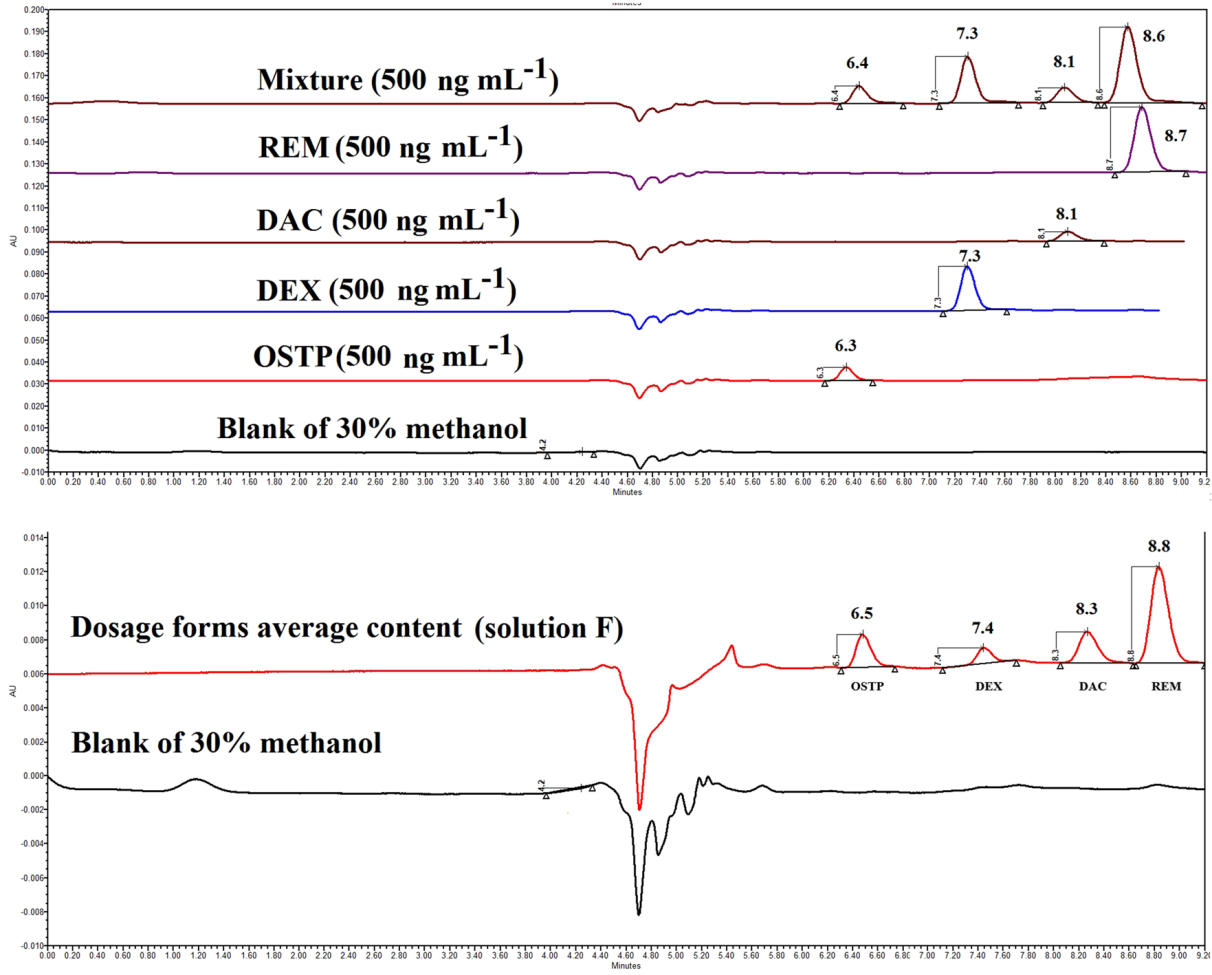
Chromatograms by the proposed method for specificity.

Blank (30.0% (v/v) methanol), OSTP, DEX, DAC, and REM were all tested separately. The acquired blank chromatograms revealed no peaks during the retention times of the drugs under consideration. Furthermore, no peaks were noticed on any of the drug chromatograms except for their own. As a consequence, they were prepared in a quaternary solution (Fig. 3). The suggested method yielded retention times of 6.323 ± 0.145, 7.166 ± 0.036, 8.078 ± 0.124, and 8.572 ± 0.166 min (eight replicates) for OSTP, DEX, DAC, and REM, respectively.

To exclude sample matrix interference, such as additives and excipients, the classical standard addition method and the innovative instrumental standard addition method were used. To the average content sample, different aliquots of a standard quaternary solution were added either manually or instrumentally. Table 3 and Table S9 demonstrated that the average content percentages obtained by the proposed method, the classic standard addition method, and the instrumental standard addition method had no difference. As a consequence, the suggested method accurately detected the examined drugs in their dosage forms even when excipients were present.

**Table 3:** Innovative instrumental standard addition.

Volume taken (µL) by UPLC from standard quaternary mixture (250 ng mL^−1^ per each drug)	Predicted amount (ng)	OSTP (%recovery)	DEX (%recovery)	DAC (%recovery)	REM (%recovery)
0	0.000	–	–	–	–
2	0.500	102.663	100.319	103.029	100.823
3	0.750	101.817	100.535	103.666	99.786
4	1.000	95.947	100.428	98.771	98.769
5	1.250	102.363	100.533	100.416	100.844
6	1.500	99.410	99.270	99.004	99.923
Intercept (a)		17,832.714	8116.500	17,516.571	78,693.786
Slope (b)		7985.943	32,557.200	12,834.114	67,790.457
Coefficient of determination (R^2^)		0.9979	0.9998	0.9988	0.9998
Predicted average content, ng		2.463	0.250	1.648	1.250
Average content (found); ng		2.233	0.249	1.521	1.161
Average content; % (by instrumental standard addition)		90.659	99.029	93.355	92.719
Average content; %^a^ (by proposed method)		91.570	99.677	92.725	91.581

^a^It is the same sample used in the innovative instrumental standard addition method.

#### Robustness and system suitability parameters

The robustness and system suitability parameters were determined both experimentally and theoretically using 2^3^ FFD equations. They were estimated experimentally by using “Eqs. (6–9)”^31^. Theoretically, they were calculated using equations derived from 2^3^ FFD estimated coefficients in uncoded units [Eqs. (S1–S12)].

6 $$k^{\prime} = \, \left( {t_{{\text{R}}} - t_{{\text{m}}} } \right)/t_{{\text{m}}}$$

where *k*′ is the analyte peak's capacity factor, commonly known as the mass distribution ratio (*D*_m_) or retention factor (*k*, *R*). Its suggested value is [0.5–10.0]^51^, where *t*_R_ is the analyte peak retention time (min) and *t*_m_ is the solvent front peak retention time (min).

7 $$\mathrm{R_{S} }= 2 (\frac{{\mathrm{Peak}}_{(\mathrm{n}+1)}{t}_{\mathrm{R}}-{{\mathrm{Peak}}_{(\mathrm{n})}t}_{\mathrm{R}}}{{W}_{(\mathrm{n}+1)}{+W}_{(n)}})$$

where the recommended value for R_S_ is greater than 1.5, which represents the resolution of two consecutive peaks (the higher R_S_, the greater the separation). Peak *t*_R_ represents peak retention time, n represents peak serial number, and W represents peak width.

8 $${\text{T }} = W_{{{5}\% }} /{2}f$$

where T is the analyte peak's tailing factor, commonly known as the symmetry factor (*As*). Its recommended range is [0.8–2]^52^. *W*_5%_ is the width of the analyte peak at 5% height, and *f* is the distance from the peak maximum to the leading edge of the peak measured at 5% of the peak height from the baseline.

9 $$\mathrm{N}= {5.54 (\frac{{t}_{\mathrm{R}}}{W50\mathrm{\%}})}$$

where the number of theoretical plates of the analyte peak, N or NTP, is recommended to be [1000–20,000] plates (the greater the N, the better the efficiency), *t*_R_ stands for retention time (min); and *W*_50%_ stands for analyte peak width at 50% height.

According to Table 4, the proposed method is robust at flow rates of [0.040–0.050], methanol percentage (v/v) ranges of [75.0%–76.0% (v/v)], and ammonium acetate concentration ranges of [8.0000 mM–8.4000 mM]. The RSD% of system suitability parameters obtained from eight chromatograms on four separate days suggested that the system was performing effectively.

**Table 4:** System suitability parameters for RP-UPLC-UV determination of oseltamivir phosphate/dexamethasone/daclatasvir dihydrochloride/remdesivir mixture.

Parameter	Oseltamivir phosphate		Dexamethasone		Daclatasvir dihydrochloride		Remdesivir	
	Factorial design value	Practical (mean ± SD^a^, RSD%^b^)	Factorial design value	Practical (mean ± SDa, RSD%b)	Factorial design value	Practical (mean ± SDa, RSD%b)	Factorial design value	Practical (mean ± SDa, RSD%^b^)
*k*′	0.584	0.526 ± 0.014, 2.591%						
R_S_			4.030	4.770 ± 0.053, 1.101%	3.373	5.402 ± 0.024, 0.447%	2.292	1.932 ± 0.056, 2.901%
T	1.854	1.633 ± 0.033, 2.037%	1.171	1.163 ± 0.003, 0.282%	1.181	1.068 ± 0.004, 0.348%	1.201	1.198 ± 0.007, 0.624%
N	7864.513	8636.185 ± 346.033, 4.007%	18,150.730	17,069.190 ± 451.941, 2.648%	17,196.340	19,916.884 ± 686.082, 3.445%	18,088.881	22,979.460 ± 82.914, 0.361%

^a^Standard deviation (4–8 replicates at three different days).

^b^Percentage relative standard deviation (4–8 replicates).

ANOVA-F at alpha = 0.05 and F-critical = 9.552 was used to compare the robustness and system suitability data obtained from the practical applications of the proposed method with those calculated from equations derived from 2^3^ FFD estimated coefficients in uncoded units. The comparison revealed no significant differences, demonstrating that factorial design may take the place of the practical method.

### Stability

According to Table 5, the UPLC vials containing laboratory-prepared standard quaternary solutions were stable in the freezer for nearly three weeks for OSTP and DEX analysis but for only about one week in the case of DAC and REM analysis.

**Table 5:** Stability of oseltamivir phosphate, dexamethasone, daclatasvir dihydrochloride, and remdesivir in freezer.

	OSTP				DEX		DAC				REM			
	10–500 (ng mL^−1^)		0.5–30 (µg mL^−1^)		50–5000 (ng mL^−1^)		25–1000 (ng mL^−1^)		0.5–25 (µg mL^−1^)		10–500 (ng mL^−1^)		0.5–30 (µg mL^−1^)	
	X (Day#)	Y^a^ = Sx/S0*100	X (Day#)	Y = Sx/S0*100	X (Day#)	Y = Sx/S0*100	X (Day#)	Y = Sx/S0*100	X (Days#)	Y = Sx/S0*100	X (Day#)	Y = Sx/S0*100	X (Day#)	Y = Sx/S0*100
X (days) versus Y% values	0	100.000	0.000	100.000	0.000	100.000	0.000	100.000	0.000	100.000	0.000	100.000	0.000	100.000
	7	100.166	4.000	100.662	7.000	100.377	7.000	100.288	4.000	100.691	7.000	103.086	4.000	101.597
	8	101.413	5.000	100.402	8.000	100.991	8.000	103.092	5.000	102.031	8.000	100.837	5.000	101.707
Stability regression equation	X = (102–99.925)/0.120		X = (102–100.040)/0.105		X = (102–99.966)/0.098		X = (102–99.830)/0.259		X = (102–99.889)/0.340		X = (102–100.165)/0.228		X = (102–100.027)/0.358	
Stability (days(X))	17.249		18.717		20.739		8.368		6.217		8.031		5.510	
Average (days(X))	**17.983**				**20.739**		**7.293**				**6.771**			
ANOVA-F^b^	**0.125**				**–**		**0.036**				**0.037**			

Significant values are in bold.

^a^(S0) represents the slope of the regression equation of the standard solutions on the first day, whereas (Sx) represents the slope of the same standard solutions on a different day.

^b^Calculated F, ANOVA test for equivalence study between proposed methods (grouped by column), ANOVA-F-critical = 7.709 (alpha = 0.05).

### Analysis of pharmaceutical dosage forms

The proposed method was successfully used to determine the average contents of OSTP, DEX, DAC, and REM in their commercial dosage forms (OSELTAMIVIR hard gelatin capsules, DEXAZONE tablets, JAVIDACLA film-coated tablets, and REMDESIVIR vials) without interference from excipients (“Selectivity and specificity” section) and in accordance with BP requirements^35^. The collected data was compared to those from previously reported techniques^8,22,26,36^ using the Student's t-test and F-Test. The comparison indicated that there was no statistically significant difference between the suggested and reported methods^8,22,26,36^ (Table S8).

According to BP standards^35^, the uniformity of the dosage unit test of each dose form was also assessed. One of two methods (weight variation or content uniformity) can be used to demonstrate the dosage unit test's uniformity. The weight variation method is applied to hard capsules or film-coated tablets containing 25 mg or more of a drug substance that comprised 25% or more, by weight, of the dosage unit. The content uniformity method is applied to solutions in unit-dose containers or uncoated/film-coated tablets containing less than 25 mg of a drug substance or comprising less than 25% by weight of the dosage unit.

The weight variation method was applied to OSELTAMIVIR hard capsules (containing 98.5 mg of OSTP and comprising 41.9% by weight of the dosage unit), while the content uniformity method was applied to DEXAZONE tablets (containing 0.5 mg of DEX and comprising less than 25 mg of a drug substance), JAVIDACLA film-coated tablets (containing 24.9% by weight of DAC in the dosage unit), and REMDESIVIR vials. The ANOVA test (P = 0.05) indicated that there was no statistically significant difference between the two obtained data sets for OSTP, DAC, and REM (Tables S1 and S12).

With such positive analytical results, the method could well be routinely used to evaluate various formulations, including the investigated drugs.

### Greenness assessment using the analytical eco-scale, the green analytical procedure index (GAPI) and AGREE method

The Analytical Eco-Scale is a great semi-quantitative tool for laboratory practice and education. However, it does not give comprehensive information regarding the protocols that have been examined^37,38^. The proposed method for the quaternary mixture was determined to be an excellent green analysis with low laboratory needs (Eco-score 78). Furthermore, the suggested method provided excellent green analysis when applied to the assessment of each drug separately (Eco-score greater than 75) (Table S13).

According to the Green Analytical Procedure Index (GAPI)^38^, the suggested method for the quaternary mixture was a direct procedure (no extraction process), as shown in Fig. S5. It also used low amounts of non-toxic chemicals and produced little waste. This was due to the low flow rate (≈ 0.050 mL min^−1^^60–62^) and environmentally friendly organic solvents. Furthermore, the suggested method was for both qualifying and quantifying. Figure S5 revealed that the suggested methods' health hazards for REM single analysis exhibited slightly toxic or slightly irritating behavior (green color). The proposed method was moderately hazardous for single analyses of OSTP, DEX, or DAC (yellow color). Short-term exposure to the solvents and chemicals used in this proposed method did not result in significant harm.

The greenness of the proposed method was also assessed by the AGREE method (Analytical GREEnness Metric Approach and Software) (Fig. S6). Analytical GREEnness is a metric system based on significance principles for assessing the greenness of analytical operations. It is comprehensive since it incorporates all 12 principles; flexible; simple to read as the result is a colorful pictogram that shows the structure of weak and strong points; and simple to apply with user-friendly GUI software. The total score is depicted in the center of the pictogram, with a number near one (0.77) and a dark green color suggesting that the assessed technique was green^39^.

## Conclusion

The suggested method is eco-friendly, sensitive, accurate, and precise, with a wide range of linearity. In terms of cost, the procedure uses inexpensive analytical reagents that are readily available in any analytical laboratory. The factorial design was used to create and optimize this procedure. The suggested method's robustness and system suitability parameters were predicted using the factorial design. The factors affecting the *k*'_(OSTP)_, T_1(OSTP)_, and R_S3(REM)_ of OSTP, DEX, DAC, and REM peaks were studied. They were affected by the interaction of three independent factors (methanol percentage, ammonium acetate concentration, and flow rate). The optimized conditions were then validated using the ICH Q2 R1 guideline. Without the influence of excipients, the proposed method was effectively used to estimate the average content and uniformity of dosage units of the drugs under study in their pharmaceutical formulations. The interference of excipients was assessed using the classic standard addition method and the innovative instrumental standard addition method. The instrumental standard addition method outperformed the classic standard addition method in that it was more accurate and sensitive, had fewer human errors, and was time-saving, economical, and more eco-friendly. The proposed method should be considered for the formulation of polypills. The developed method has high sensitivity, so it is suitable for assaying in human plasma. Additionally, the analytical Eco-Scale, GAPI, and AGREE proved the greenness of the proposed method, which aids its utility in routine applications.

## Supplementary Information

Supplementary Information.

## Data Availability

The datasets generated and/or analyzed during the current study are available from the corresponding author on reasonable request.
