# Supplementary material for: Development of UPLC method for simultaneous assay of some COVID-19 drugs utilizing novel instrumental standard addition and factorial design
Source: Sci Rep. 2023 Apr 4;13:5466. doi: 10.1038/s41598-023-32405-x (PMC10071232; doi:10.1038/s41598-023-32405-x)
Supplement: Supplementary file 1 — Supplementary Information. [file 41598_2023_32405_MOESM1_ESM.pdf]

Development of UPLC method for simultaneous assay of some COVID-19 drugs utilizing novel instrumental standard addition and factorial design

Hanan I. EL-Shorbagy[*, 1], Mona A. Mohamed[2], Alaa El-Gindy[1], Ghada M. Hadad[1], Fathalla Belal[3].

[1] Pharmaceutical Analytical Chemistry Department, Faculty of Pharmacy, Suez Canal University, Ismailia 41522, Egypt.

[2] Pharmaceutical Chemistry Department, Egyptian Drug Authority (EDA), Cairo, Egypt.

[3] Department of Pharmaceutical Analytical Chemistry, Faculty of Pharmacy, Mansoura University, Mansoura 35516, Egypt.

* Corresponding author. PGS.202214@pharm.suez.edu.eg & Hananibrahimelshorbagy@gmail.com.

Table of supplementary material figure captions:

Fig. S1: Molecular structures and IUBAC names of (A) oseltamivir phosphate, (B) dexamethasone, (C) daclatasivir dihydrochloride, and (D) remdesivir.

Fig. S2: PDA scans (200-400 nm) of oseltamivir phosphate, dexamethasone, daclatasivir dihydrochloride, and remdesivir.

Fig. S3: The evaluated wave lengths during 2^3 FFD experiments of the analyzed drugs.

Fig. S4: Overlay of the chromatograms of oseltamivir phosphate/dexamethasone/daclatasvir dihydrochloride/remdesivir mixures within the linearity ranges using the developed method.

Fig. S5: GAPI assessment of the green profile of: A- the proposed method and single analyses of OSTP, DEX, or DAC. B- REM single analysis.

Fig. S6: Result of AGREE analysis for the proposed method.

Fig. S1

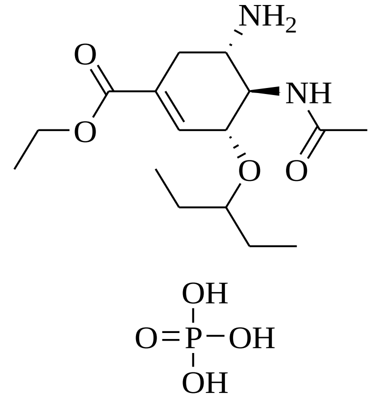

A

Ethyl (3R,4R,5S)-4-acetamido-5-amino-3-pentan-3-yloxycyclohexene-1-carboxylate;phosphoric acid

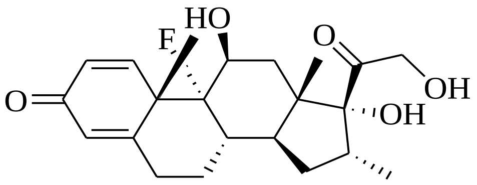

B

(8S,9R,10S,11S,13S,14S,16R,17R)-9-fluoro-11,17-dihydroxy-17-(2-hydroxyacetyl)-10,13,16-trimethyl-6,7,8,11,12,14,15,16-octahydrocyclopenta[a]phenanthren-3-one

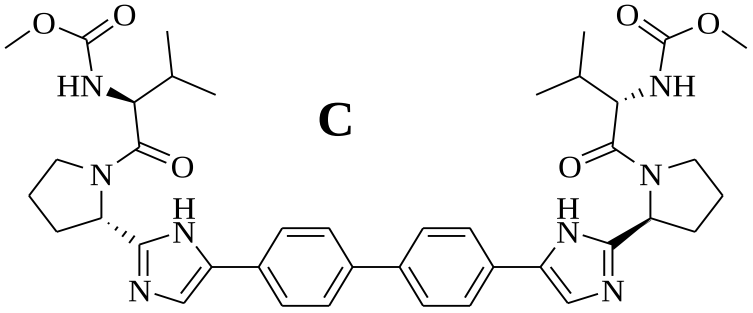

C

HCHCl

Methyl N-[(2S)-1-[(2S)-2-[5-[4-[4-[2-[(2S)-1-[(2S)-2-(methoxycarbonylamino)-3-methylbutanoyl]pyrrolidin-2-yl]-1H-imidazol-5-yl]phenyl]phenyl]-1H-imidazol-2-yl]pyrrolidin-1-yl]-3-methyl-1-oxobutan-2-yl]carbamate;dihydrochloride

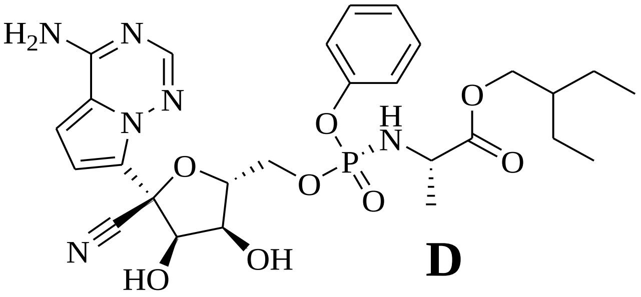

D

2-ethylbutyl (2S)-2-[[[(2R,3S,4R,5R)-5-(4-aminopyrrolo[2,1-f][1,2,4]triazin-7-yl)-5-cyano-3,4-dihydroxyoxolan-2-yl]methoxy-phenoxyphosphoryl]amino]propanoate

Fig. S2

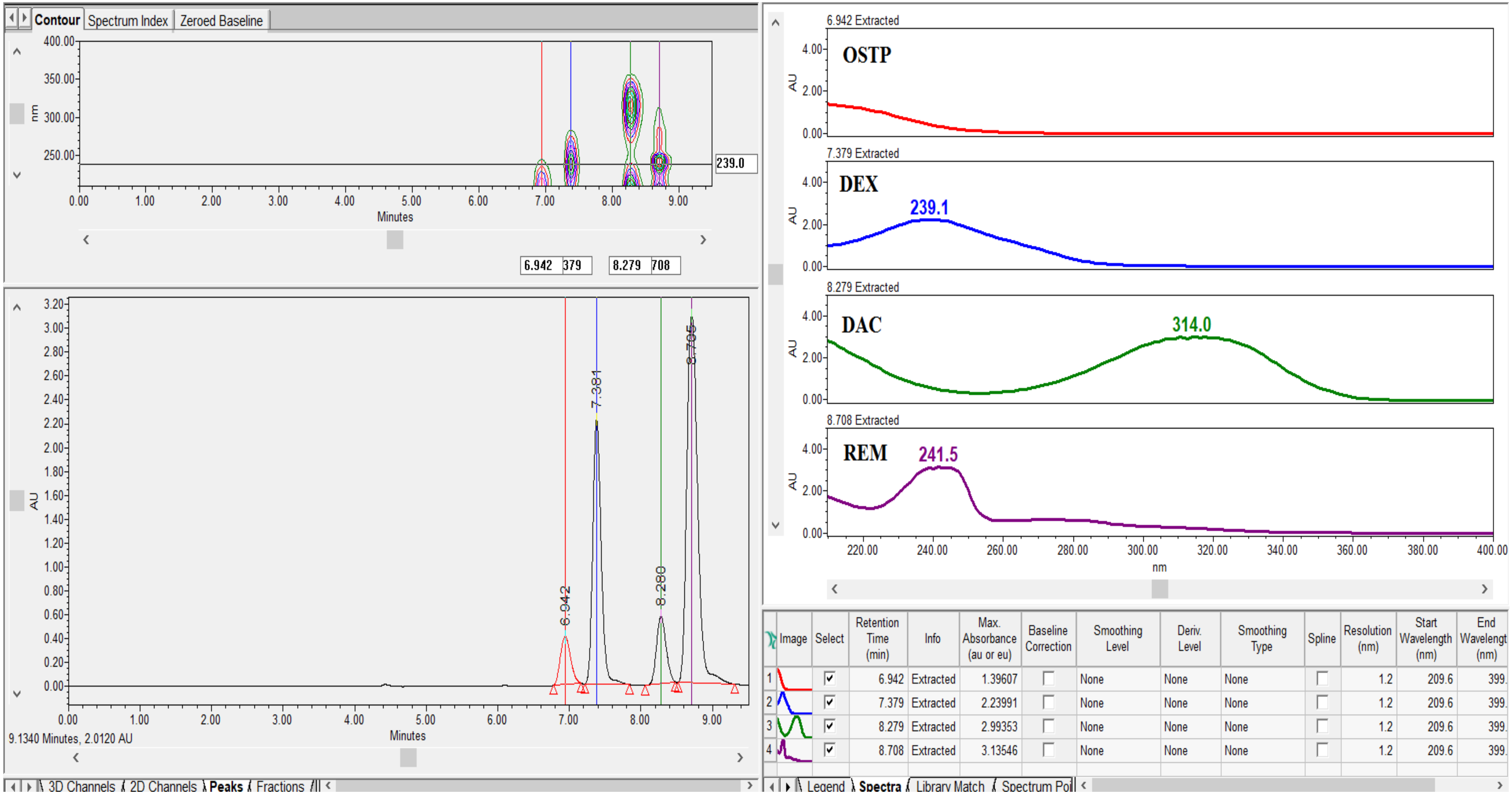
Contour
Spectrum Index
Zeroed Baseline
nm
Minutes
239.0
6.942
379
8.279
708
AU
7.381
6.942
8.280
8.705
9.1340 Minutes, 2.0120 AU
3D Channels
2D Channels
Peaks
Fractions
6.942 Extracted
OSTP
7.379 Extracted
DEX
239.1
8.279 Extracted
DAC
314.0
8.708 Extracted
REM
241.5
nm
Image
Select
Retention Time (min)
Info
Max. Absorbance (au or eu)
Baseline Correction
Smoothing Level
Deriv. Level
Smoothing Type
Spline
Resolution (nm)
Start Wavelength (nm)
End Wavelengt (nm)
1 6.942 Extracted 1.39607 None None None 1.2 209.6 399.
2 7.379 Extracted 2.23991 None None None 1.2 209.6 399.
3 8.279 Extracted 2.99353 None None None 1.2 209.6 399.
4 8.708 Extracted 3.13546 None None None 1.2 209.6 399.
Legend
Spectra
Library Match
Spectrum Poi

Fig. S3

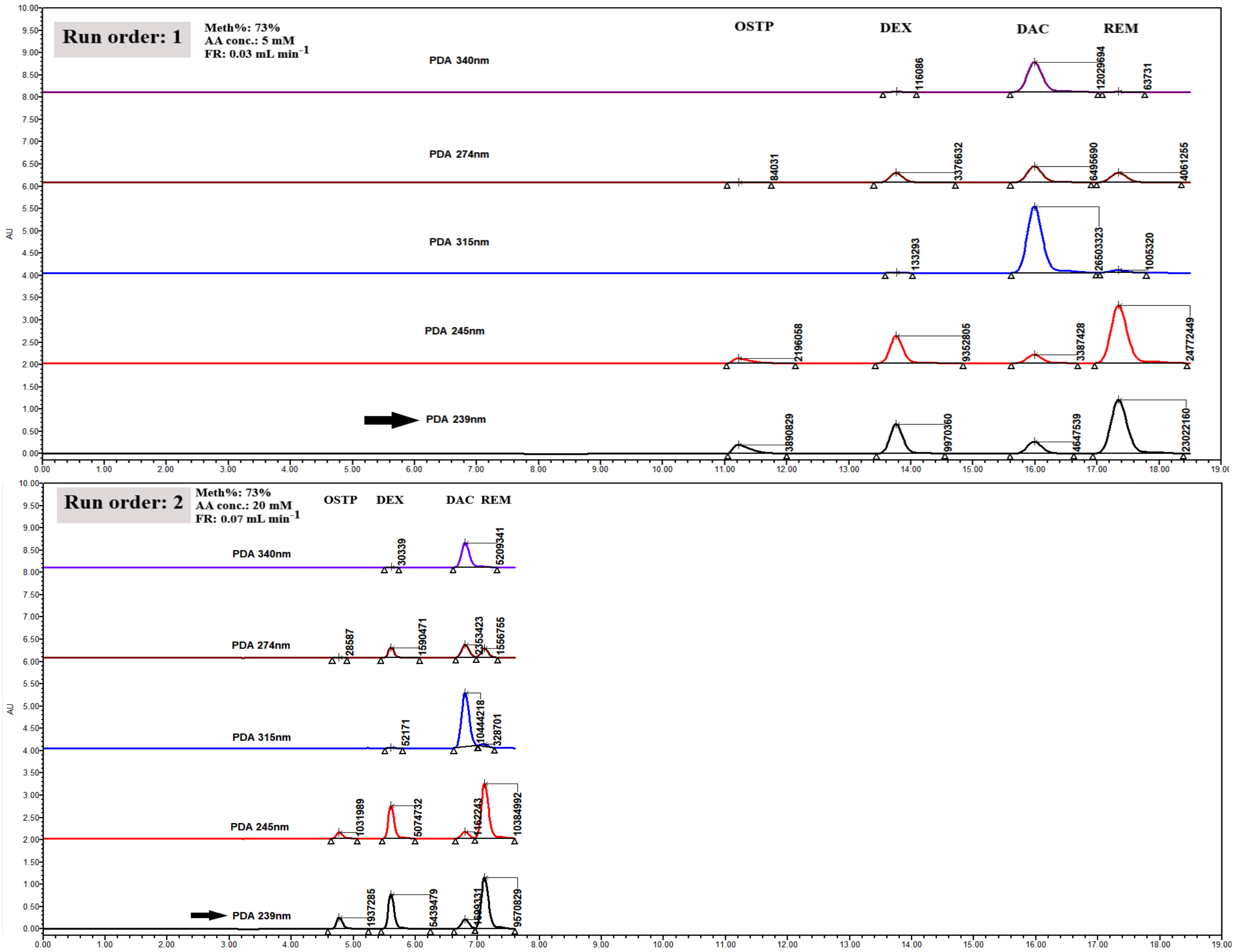
Run order: 1
Meth%: 73%
AA conc.: 5 mM
FR: 0.03 mL min-1
OSTP
DEX
DAC
REM
PDA 340nm
PDA 274nm
PDA 315nm
PDA 245nm
PDA 239nm
AU
Run order: 2
Meth%: 73%
AA conc.: 20 mM
FR: 0.07 mL min-1
OSTP
DEX
DAC
REM
PDA 340nm
PDA 274nm
PDA 315nm
PDA 245nm
PDA 239nm
AU

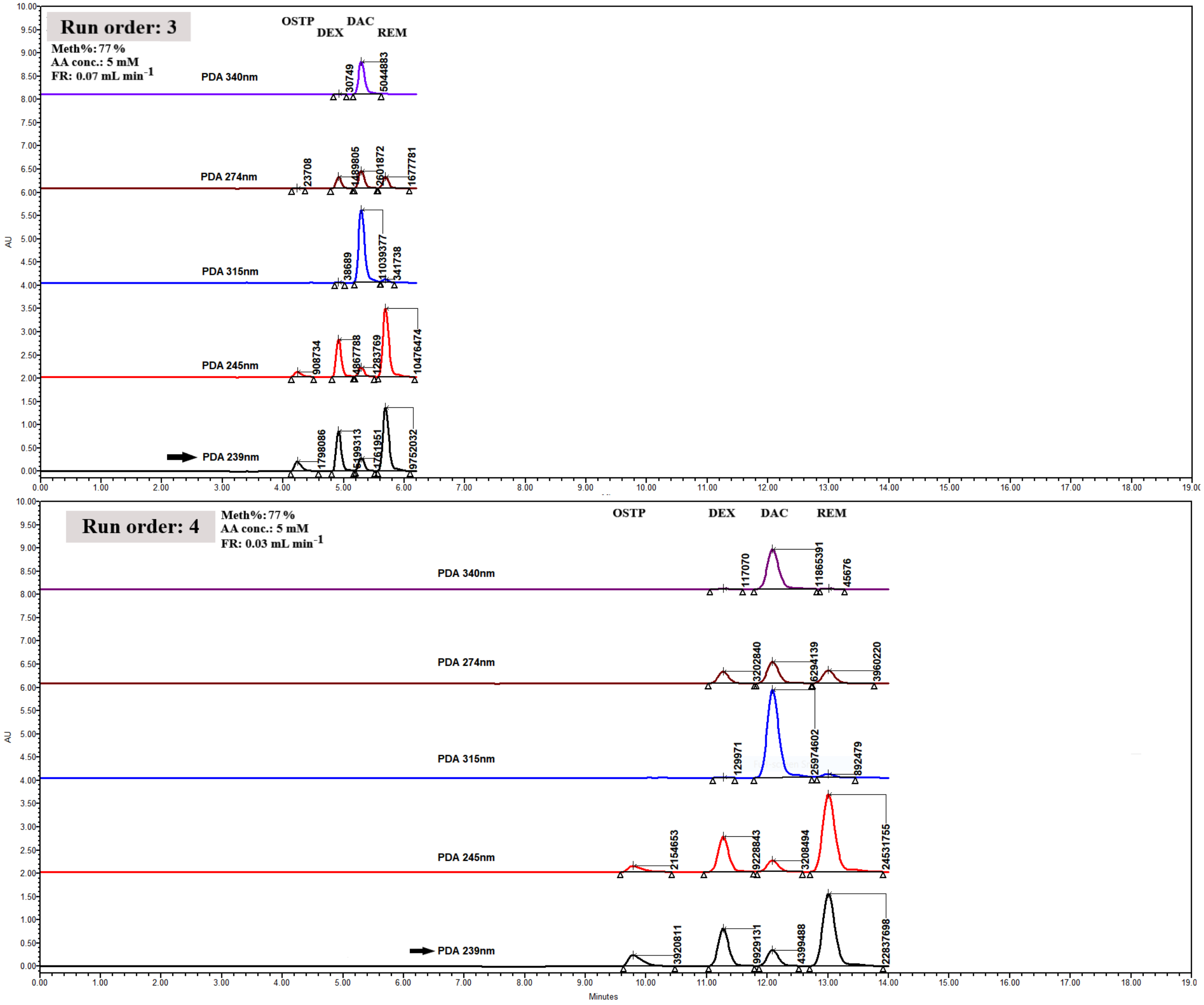
Run order: 3
Meth%: 77 %
AA conc.: 5 mM
FR: 0.07 mL min-1
OSTP
DEX
DAC
REM
PDA 340nm
PDA 274nm
PDA 315nm
PDA 245nm
PDA 239nm
Run order: 4
Meth%: 77 %
AA conc.: 5 mM
FR: 0.03 mL min-1
AU
Minutes

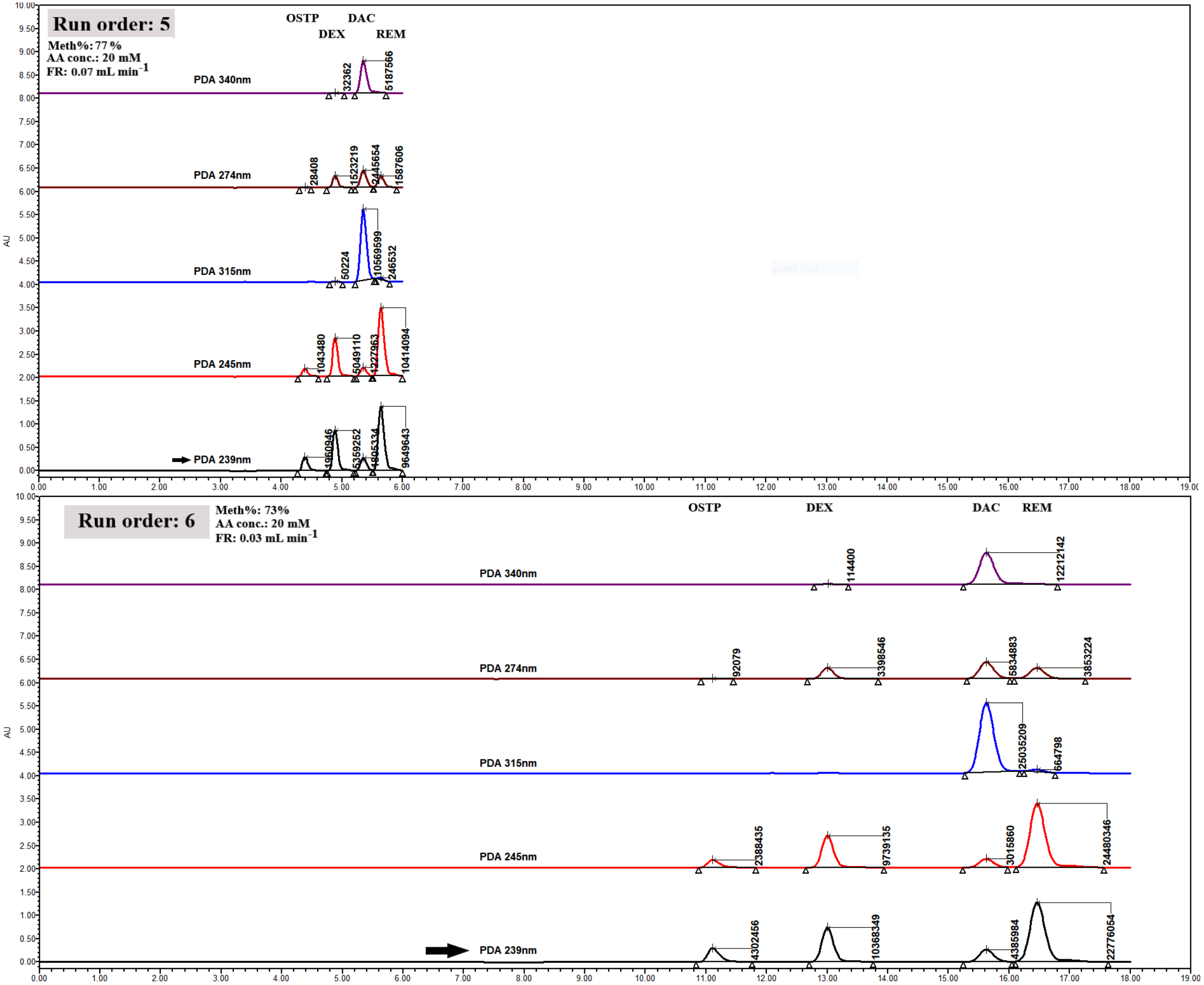

Run order: 5
Meth%: 77 %
AA conc.: 20 mM
FR: 0.07 mL min-1
OSTP
DAC
DEX
REM
PDA 340nm
32362
5187566
PDA 274nm
28408
1523219
2445654
1587606
PDA 315nm
50224
10569599
246532
PDA 245nm
1043480
5049110
4227963
10414094
PDA 239nm
1960946
5359252
1805334
9649643
AU
Run order: 6
Meth%: 73%
AA conc.: 20 mM
FR: 0.03 mL min-1
OSTP
DEX
DAC
REM
PDA 340nm
114400
12212142
PDA 274nm
92079
3398546
5834883
3853224
PDA 315nm
25035209
664798
PDA 245nm
2388435
9739135
3015860
24480346
PDA 239nm
4302456
10368349
4385984
22776054
AU

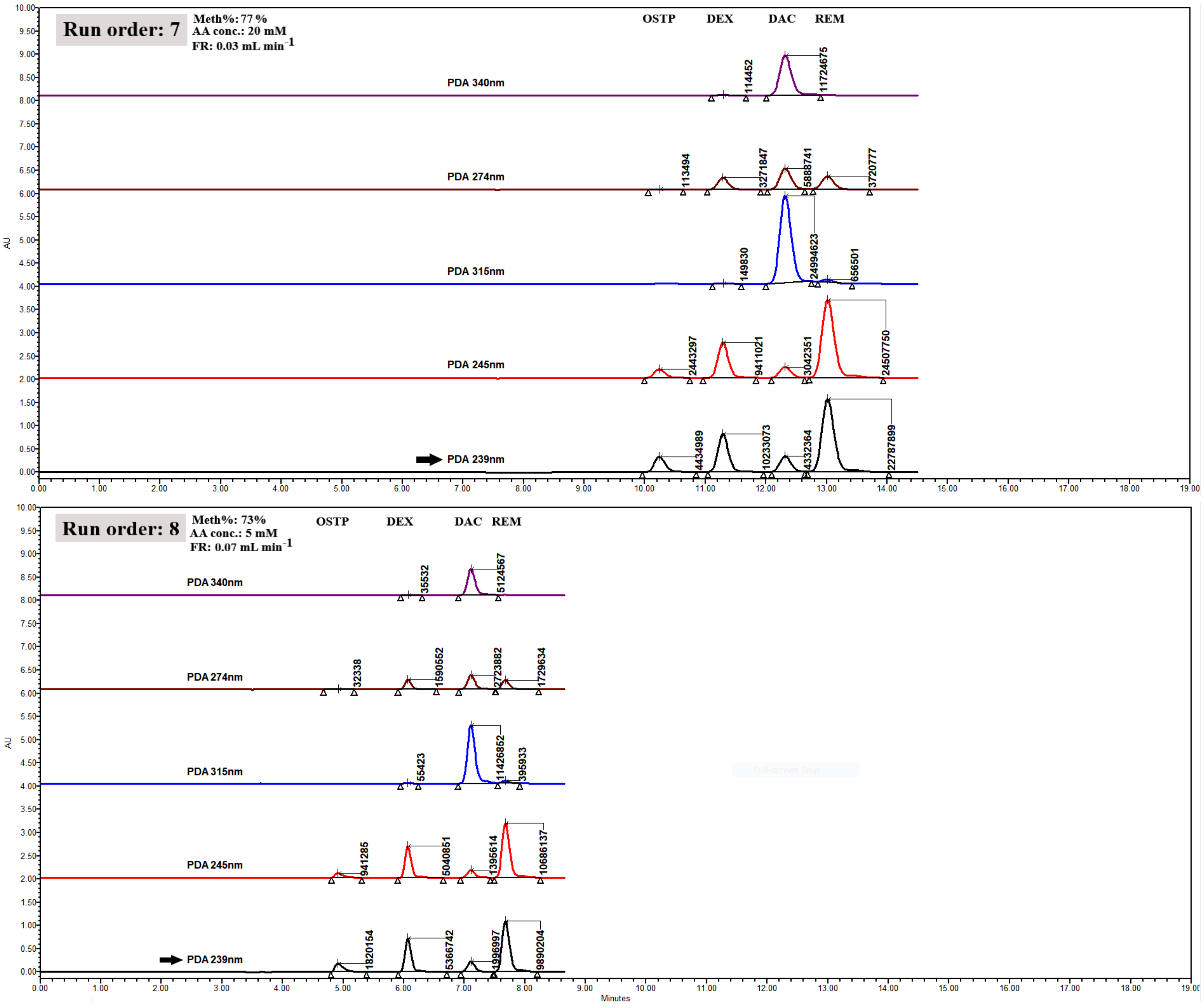
Run order: 7
Meth%: 77 %
AA conc.: 20 mM
FR: 0.03 mL min^{-1}
OSTP
DEX
DAC
REM
PDA 340nm
114452
11724675
PDA 274nm
113494
3271847
5888741
3720777
PDA 315nm
149830
24994623
656501
PDA 245nm
2443297
9411021
3042351
24507750
PDA 239nm
4434989
10233073
4332364
22787899
AU
Run order: 8
Meth%: 73%
AA conc.: 5 mM
FR: 0.07 mL min^{-1}
OSTP
DEX
DAC
REM
PDA 340nm
35532
5124567
PDA 274nm
32338
1590552
2723882
1729634
PDA 315nm
55423
11426852
395933
PDA 245nm
941285
5040851
1395614
10686137
PDA 239nm
1820154
5366742
1996997
9890204
AU
Minutes

Fig. S4

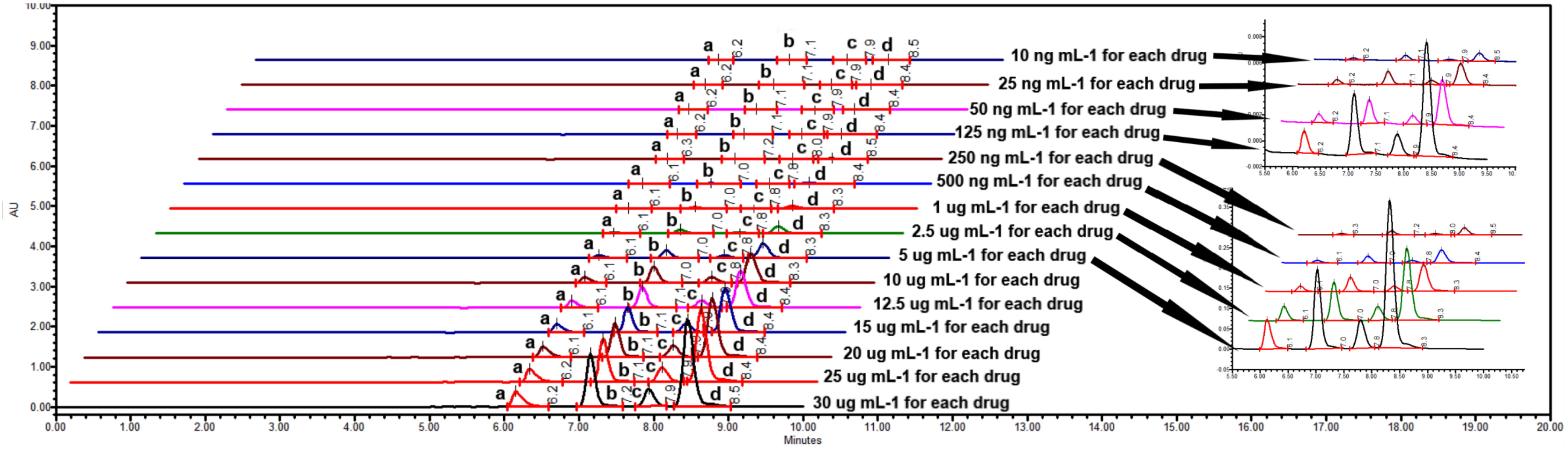
10 ng mL-1 for each drug
25 ng mL-1 for each drug
50 ng mL-1 for each drug
125 ng mL-1 for each drug
250 ng mL-1 for each drug
500 ng mL-1 for each drug
1 ug mL-1 for each drug
2.5 ug mL-1 for each drug
5 ug mL-1 for each drug
10 ug mL-1 for each drug
12.5 ug mL-1 for each drug
15 ug mL-1 for each drug
20 ug mL-1 for each drug
25 ug mL-1 for each drug
30 ug mL-1 for each drug
AU
Minutes

Fig. S5

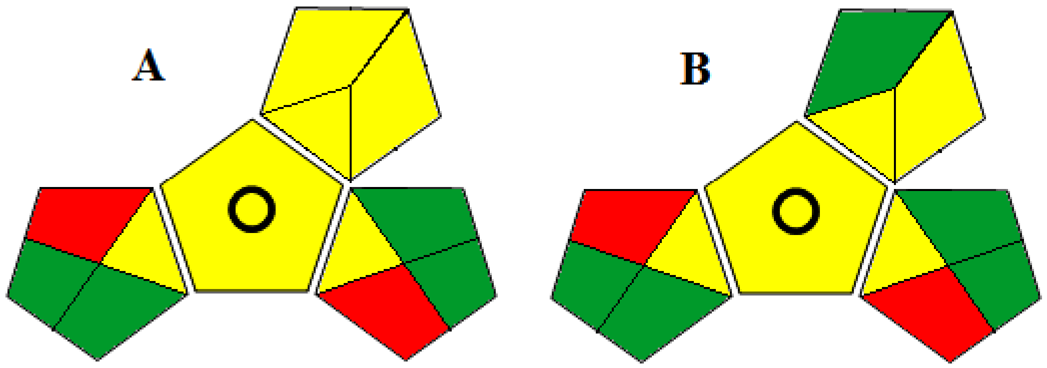

Fig. S6

Analytical Greenness report sheet

29/07/2022 21:20:01

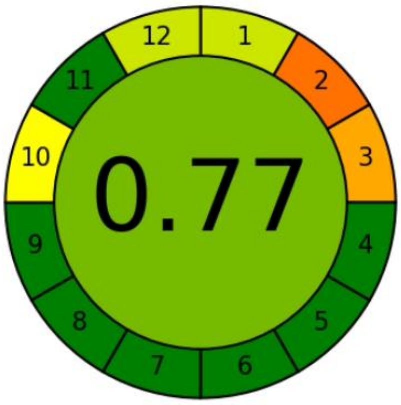

Criteria	Score	Weight
1. Direct analytical techniques should be applied to avoid sample treatment.	0.6	2
2. Minimal sample size and minimal number of samples are goals.	0.22	2
3. If possible, measurements should be performed in situ.	0.33	2
4. Integration of analytical processes and operations saves energy and reduces the use of reagents.	1.0	2
5. Automated and miniaturized methods should be selected.	1.0	2
6. Derivatization should be avoided.	1.0	2
7. Generation of a large volume of analytical waste should be avoided, and proper management of analytical waste should be provided.	1.0	2
8. Multi-analyte or multi-parameter methods are preferred versus methods using one analyte at a time.	1.0	2
9. The use of energy should be minimized.	1.0	2
10. Reagents obtained from renewable sources should be preferred.	0.5	2
11. Toxic reagents should be eliminated or replaced.	1.0	2
12. Operator's safety should be increased.	0.6	2

Table S1: Response optimization and optimization plot of 2^3 full factorial design for RP-UPLC-PDA separation of oseltamivir phosphate/dexamethasone/daclatasivir dihydrochloride/remdesivir mixture.

Response optimization

	Goal	Lower	Target	Weight	Import	Predicted Responses	Desirability (d)
$k'_{(OSTP)}$	Maximize	0.5	0.55	1	1	0.5833	1
$T_{1(OSTP)}$	Maximize	1.5	1.8	1	1	1.8531	1
$Rs_{3(REM)}$	Maximize	2.0	2.3	1	1	2.3007	1

Optimum Condition:

Methanol% = 75.7071%

Ammonium acetate (mM) = 8.1818 mM

Flow rate (mL min^{-1}) = 0.0482 mL min^{-1}

Composite Desirability (D) = 1

Optimization plot

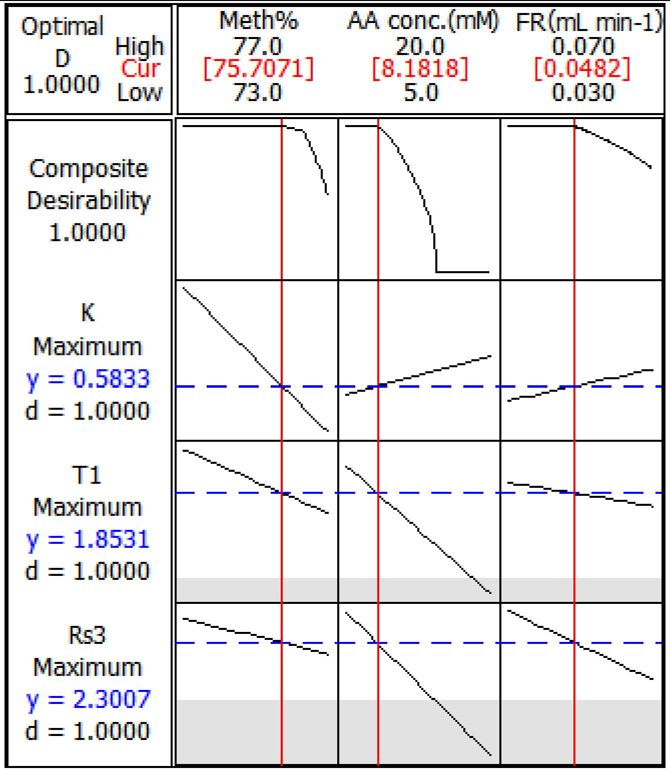

Table S2: Method development trials by RP-UPLC-PDA

Day #	Trial #	Method #	Drug (µg mL^{-1})	Injection volume (µL)	Methods' condition						Results							Observation	Recommendation
					Column temperature	Methanol%	Aqueous mobile phase %	Flow rate (mL min^{-1})	PDA (nm)	Diluting solvent	Peaks Rt (min)	W (min)	Tusp	N	*K'*	R_S	AUC		
1	1	1	REM (100)	10	-	50	50 water	0.2	240	60% methanol	37.5 min	0.244	1.1	>19000	43.6	-	17848043	Deacrease K	Use ACN instesd of methanol & 0.1% acetic acid or amm. acetate instesd of water
1	2	1	OSTP (100)	10	-	50	50 water	0.2	240	50% methanol	15.197 min	15.759	7.7	>29	13.8	-	1317827	Decrease W,K and increase N	
2	3	1	OSTP (100)	10	-	50	50 water	0.2	240	50% methanol	2 Peaks at 1.4 and 15.87 min	Peak1 =0.253 & peak 2= 14.386	1.5 & 5.6	6000 & 39	0.789	-	432739 & 800174	It may be unstable	protect samples from light
2	4	2	REM (100)	10	-	60	40 water	0.2	240	50% methanol	peak at 7.906	0.23	1.12	17354	8.9		18389847	increasing methanol % decreased rt from 37.5 to 7.9 (compare method 4 with 2)	
2	5	2	OSTP (330)	5	-	60	40 water	0.2	240	50% methanol	6.333	14.1	6.26	13	6.9	-	2203105	decrease rt FROM 15 TO 6.3 MIN (compare method 4 with 2) (OSTP WAS he main proplem)	I tried 5% ACN taken from water& FR 0.15
3	6	3	OSTP (330)	5	-	70	30 Water	0.1	240	50% methanol	8.817	15.164	4.7	20	3.4085	-	4552702	no improvemen (compare method 9 with 8)	remove ACN
3	7	4	OSTP (330)	5	-	80	20 Water	0.2	240	50% methanol	3.319	4.541	5.4	19	3.14875	-	2412515	COMPARING method 10 with methods2&4 : decrease in rt,K,W,T& bad N &	I used 0.1% acetic acid (Ph3.3) instead of water (ph6.8)

																	increae in AUC	
3	8	5	OSTP (330)	5	-	80	20 .1% acetic acid	0.2	24 0	50% methanol	5.37	4.888	0.58	25	5.7125	-	1587850	Forked peak was developed(c ompare method 11 with 10)	I increased 0.1% acetic acid (Ph3.3) and decreased FR
3	9	6	OSTP (330)	5	-	70	30 .1% acetic acid	0.1 5	24 0	50% methanol	2.103	7.136	1.3	26	1.103	-	1926119		
3	10	7	OSTP (330)	5	-	50	50 .1% acetic acid	0.1 5	24 0	50% methanol	3.094	2.816	1.2	70	2.094	-	3318620	comparing method 15 with 12: general improvemen t in syste suitability parameters (N is small and W is high)	INCREASE METHAN OL
4	11	7	OSTP (75)	5	-	50	50 .1% acetic acid	0.1 5	24 0	100% water	3.707	1.934	1.02	136	2.707	-	1291868		Try ammonium acetate to mask silanol groups to decrease W and Increase T).
4	12	7	OSTP (75)	5	-	50	50 .1% acetic acid	0.1 5	24 0	100% water	4.127	2.098	1.07	130	2.098	-	1284948		
4	13	8	OSTP (75)	5	25	50	50 10m M AA	0.1 5	24 0	100% water	3.883	1.932	1.03	134	2.883	-	1293009		decreae AA
4	14	9	OSTP (75)	5	25	55	45 10m M AA	0.1 5	24 0	100% water	3.192	0.57	2.2	4741	2.192	-	1424364	N increased, W decreased but T increased	
4	15	9	OSTP (75)	2.5	25	55	45 10m M AA	0.1 5	24 0	100% water	3.973	0.676	2.07	6752	2.973	-	723748		
5	16	9	OSTP (11)	10	25	55	45 10m M AA	0.1 5	24 0	100% water	5.102	1.019	0.7	3455	4.102	-	244673		Increase methanol
5	17	10	OSTP (75)	10	25	60	40 10m M AA	0.1 5	24 0	100% water	3.734	0.795	1.9	1500	2.734	-	3022628		Inject REM
5	18	10	REM (100)	10	25	60	40 10m M AA	0.1 5	24 0	60% methanol	11.097	1	1.07	19788	10.097	-	25692780		Inject DEXP
6	19	10	Dexamethaso ne phosphate	10	25	60	40 10m M AA	0.1 5	24 0	Water	2.785	0.925	2.3	3668	1.78	-	864263	Large peak good system sutbility parameters	Inject mix of DEXP, OSTP &REM
6	20	10	DEXP+OST	10	25	60	40	0.1	24	Water	DEXP(2.79	DEXP(0.723),OS	DEXP(	DEXP(43	DEXP(1.795	R1(5.	DEXP(312	DEXP had	Increase

			P+REM				10mM AA	5	0		5), OSTP(3.681), REM(10.471)	TP(0.3), REM(0.627)	2.2), OSTP(1.7) REM(1.07)	02), OSTP(14061), REM(20265)	)	7), R2(31.8)	9249), OSTP(718604), REM(1130915)	high T	AA conc.
7	21	10	DEXP (50) + OSTP (45) + REM (50)	10	25	60	40 20mM AA	0.15	240	5% methanol	DEXP(3.077), OSTP(3.706), REM(10.974)	DEXP(0.661),OSTP(0.513), REM(0.67)	DEXP(2.5), OSTP(1.8) REM(1.08)	DEXP(4161), OSTP(15451), REM(20268)	DEXP(2.077)	R1(3.7), R2(33.3)	DEXP(5762374), OSTP(908178), REM(3516146)	DEXP had higher T	Increase flow rate
7	22	11	DEXP (50) + OSTP (45) + REM (50)	10	25	60	40 20mM AA	0.2	240	5% methanol	DEXP(2.324), OSTP(2.793), REM(8.268)	DEXP(0.455),OSTP(0.374), REM(0.763)	DEXP(2.5), OSTP(1.7) REM(1.08)	DEXP(4016), OSTP(14544), REM(17996)	DEXP(1.905)	R1(3.7), R2(31.6)	DEXP(4417072), OSTP(677769), REM(2630649)	T of DEXP was still high	Increase methanol% with decreasing FR gradually
7	23	12	DEXP (50) + OSTP (45) + REM (50)	10	25	65	35 20mM AA	0.1	240	5% methanol	DEXP(3.578), OSTP(4.371), REM(9.13)	DEXP(0.584),OSTP(0.395), REM(0.59)	DEXP(2.0), OSTP(1.6) REM(1.08)	DEXP(6499), OSTP(18053), REM(23902)	DEXP(0.789)	R1(4.9), R2(25.4)	DEXP(8366167), OSTP(1361413), REM(5289580)		
7	24	13	DEXP (50) + OSTP (45) + REM (50)	10	25	70	30 20mM AA	0.08	240	5% methanol	DEXP(3.852), OSTP(4.646), REM(7.434)	DEXP(0.6),OSTP(0.397), REM(0.436)	DEXP(1.8), OSTP(1.4) REM(1.09)	DEXP(9059), OSTP(20155), REM(25634)	DEXP(0.605)	R1(5.2), R2(17.2)	DEXP(1010775), OSTP(1736506), REM(6633015)		
7	25	14	DEXP (50) + OSTP (45) + REM (50)	10	25	75	25 20mM AA	0.075	240	5% methanol	DEXP(3.64), OSTP(4.324), REM(5.71)	DEXP(0.631),OSTP(0.387), REM(0.388)	DEXP(1.9), OSTP(1.3) REM(1.1)	DEXP(10658), OSTP(19853), REM(24509)	DEXP(0.456)	R1(5.0), R2(10.1)	DEXP(1052005), OSTP(1860399), REM(7089030)		
7	26	15	DEXP (50) + OSTP (45) + REM (50)	10	25	75	25 20mM AA	0.07	240	5% methanol	DEXP(3.861), OSTP(4.588), REM(6.001)	DEXP(0.662),OSTP(0.298), REM(0.396)	DEXP(2.0), OSTP(1.2) REM(1.1)	DEXP(10787), OSTP(20084), REM(24458)	DEXP(0.379)	R1(5.0), R2(9.7)	DEXP(1108159), OSTP(1965220), REM(7570356)		
7	27	16	DEXP (50) + OSTP (45) + REM (50)	10	25	77	23 20mM AA	0.07	240	5% methanol	DEXP(3.786), OSTP(4.457), REM(5.55)	DEXP(0.629),OSTP(0.266), REM(0.409)	DEXP(2.0), OSTP(1.2) REM(1.1)	DEXP(11180), OSTP(20613), REM(25225)	DEXP(0.352)	R1(4.8), R2(8.1)	DEXP(1087413), OSTP(1950784), REM(7594206)		
8	28	17	DEXP (50) + OSTP (45) + REM (50)	1	25	77	23 10mM AA	0.07	240	5% methanol	DEXP(3.518), OSTP(4.307), REM(5.38)	DEXP(0.678),OSTP(0.272), REM(0.434)	DEXP(2.9), OSTP(1.3) REM(1.1)	DEXP(3783), OSTP(15747), REM(21034)	DEXP(0.256)	R1(4.0), R2(7.3)	DEXP(867327), OSTP(188469), REM(792860)		
8	29	17	DEXP (50) +	0.1	25	77	23 10m	0.07	240	5% methanol	DEXP(3.549),	DEXP(0.764),OSTP(0.26),	DEXP(2.3),	DEXP(911),	DEXP(0.267 MAIN)	R1(2.2),	DEXP(63049),	Low k’ and high T	

			OSTP (45) + REM (50)				M AA				OSTP(4.255), REM(5.27)	REM(0.401)	OSTP(1.4) REM (1.1)	OSTP(12802), REM(16338)		R2(6.3)	OSTP(15595), REM(75054)		
9	30	17	DEX (4)	10	25	77	23 10mM AA	0.07	240	100%methanol	4.743	0.358	1.2	18208	0.693928571	-	18674087		
9	31	17	DEX (4)	10	25	77	23 10mM AA	0.07	240	Water	4.711	0.35	1.1	25233	0.6825	-	1195128		
9	32	17	DEX (4)	10	25	77	23 10mM AA	0.07	240	Mobile phase	4.706	0.35	1.1	25101	0.680714286	-	1253401		Inject mixture of DEX, OSTP &REM in mobile phase
9	33	17	OSTP (5) + DEX (4) + REM (5)	10.0	25	77	23 10mM AA	0.07	240	Mobile phase	OSTP(4.377,4.471 FORKED PEAK), DEX(4.661), REM(5.424)							Baseline was so noisy	Inject mixture of DEX, OSTP &REM in water
9	34	17	OSTP (5) + DEX (4) + REM (5)	10.0	25	77	23 10mM AA	0.07	240	Water	OSTP(4.414), DEX(4.703), REM(5.457)	OSTP(0.254),DEX(0.432), REM(0.44)	OSTP(1.5), DEX(1.1) REM (1.1)	OSTP(18033), DEX(25028) REM (23323)	0.576428571	R1(2.2), R2(5.6)	OSTP(102088), DEX(1268185) REM (226790)		
9	35	17	OSTP (5) + DEX (4) + REM (5)	5.0	25	77	23 10mM AA	0.07	240	Water	OSTP(4.333), DEX(4.613), REM(5.359)	OSTP(0.255),DEX(0.337), REM(0.3)	OSTP(1.3), DEX(1.1) REM (1.1)	OSTP(16419), DEX(23777) REM (22322)	0.5475	R1(2.1), R2(5.5)	OSTP(51200), DEX(616040) REM (111380)		Try DAC
10	36	17	DAC (100)	10	25	77	23 10mM AA	0.07	240	10% methanol	5.252	0.426	1.1	18509	0.875714286	-	4773080		
10	37	17	DAC (100)	1	25	77	23 10mM AA	0.07	240	10% methanol	5.073	0.465	1.1	16487	0.811785714	-	488529		
10	38	17	DAC (100)	0.1	25	77	23 10mM AA	0.07	240	10% methanol	5.04	0.357	1.1	12560	0.8	-	44880		INJECT MIXture f OSTP, DEX, DAC AND REM
11	39	17	OSTP (50) + DEX (50) + DAC (50)	1	25	77	23 10mM	0.07	240	10% methanol	OSTP(4.315), DEX(4.598)	OSTP(0.260), DEX(0.289), DAC(0.243),	OSTP(1.2), DEX(1.	OSTP(15247), DEX(215	0.541071429	R1(2.0), R2(3.	OSTP(244504), DEX(1485		

			+REM (50)				AA				, DAC(5.129), REM(5.403)	REM(0.359)	1), DAC(1.1), REM(1.1)	63), DAC(16801), REM(21049)		6), R3(1.7)	427), DAC(399709), REM(2442903)		
11	40	17	OSTP (50) + DEX (50) + DAC (50) +REM (50)	0.1	25	77	23 10mM AA	0.07	240	10% methanol	OSTP(4.295), DEX(4.564), DAC(5.087), REM(5.363)	OSTP(0.268), DEX(0.287), DAC(0.294), REM(0.356)	OSTP(1.1), DEX(1.1), DAC(1.0), REM(1.1)	OSTP(11745), DEX(15689), DAC(12842), REM(16079)	0.533928571	R1(1.7), R2(3.1), R3(1.5)	OSTP(22387), DEX(135823), DAC(37208), REM(226972)		DECREAE METHANOL TO INCREASE R
11	41	18	OSTP (50) + DEX (50) + DAC (50) +REM (50)	1	25	75	25 10mM AA	0.07	240	10% methanol	OSTP(4.509), DEX(4.89), DAC(5.716), REM(5.995)	OSTP(0.323), DEX(0.365), DAC(0.281), REM(0.499)	OSTP(1.3), DEX(1.1), DAC(1.0), REM(1.1)	OSTP(15289), DEX(22025), DAC(16420), REM(21772)	0.610357143	R1(2.6), R2(5.2), R3(1.6)	OSTP(249281), DEX(1486154), DAC(398764), REM(2409176)		Decrease flow rate to increase R
11	42	19	OSTP (50) + DEX (50) + DAC (50) +REM (50)	1	25	75	25 10mM AA	0.05	240	10% methanol	OSTP(6.319), DEX(6.846), DAC(8.035), REM(8.457)	OSTP(0.383), DEX(0.394), DAC(0.368), REM(0.579)	OSTP(1.2), DEX(1.0), DAC(1.0), REM(1.0)	OSTP(16745), DEX(23760), DAC(18479), REM(23276)	0.50452381	R1(2.7), R2(5.6), R3(1.8)	OSTP(342052), DEX(1924705), DAC(567870), REM(3422102)		
12	43	20	OSTP (50) + DEX (50) + DAC (50) +REM (50)	10	25	70	30 5mM AA	0.03	239	Dilution with water	OSTP(11.454), DEX(12.581), DAC(16.337), REM(17.096)	OSTP(0.964), DEX(0.883), DAC(0.812), REM(0.983)	OSTP(1.0), DEX(1.0), DAC(0.9), REM(1.0)	OSTP(8090), DEX(17734), DAC(14126), REM(15897)	0.735454545	R1(2.5), R2(7.9), R3(1.3)	OSTP(7222295), DEX(28534465), DAC(8388240), REM(25699908)		
12	44	21	OSTP (50) + DEX (50) + DAC (50) +REM (50)	10	25	73	27 5mM AA	0.08	239	Dilution with water	OSTP(4.264), DEX(4.629), DAC(5.631), REM(5.853)	OSTP(0.364), DEX(0.370), DAC(0.277), REM(0.313)	OSTP(1.1), DEX(1.0), DAC(0.8), REM(1.1)	OSTP(10524), DEX(18865), DAC(15670), REM(18936)	0.496140351	R1(2.3), R2(6.3), R3(1.2)	OSTP(2778221), DEX(12579139), DAC(2703204), REM(9938800)		
12	45	22	OSTP (50) + DEX (50) + DAC (50) +REM (50)	10	25	73	27 5mM AA	0.03	239	Dilution with water	OSTP(11.67), DEX(12.658), DAC(15.442), REM(16.084)	OSTP(0.863), DEX(0.861), DAC(0.755), REM(0.879)	OSTP(1.0), DEX(1.0), DAC(0.9), REM(1.0)	OSTP(10654), DEX(23341), DAC(20160), REM(21739)	0.721969697	R1(2.3), R2(6.7), R3(1.6)	OSTP(7273480), DEX(28991205), DAC(9285770), REM(28545648)		increase methanol% in the solvent to prevent drug adsorption during filtration.
12	46	23	OSTP (50) +	10	25	73	27 5mM	0.07	239	Dilution with	OSTP(4.906),	OSTP(0.401), DEX(0.357),	OSTP(1.1),	OSTP(11209),	0.738947368	R1(2.4),	OSTP(3139964),		

			DEX (50) + DAC (50) +REM (50)				AA			water	DEX(5.326), DAC(6.491), REM(6.744)	DAC(0.274), REM(0.329)	DEX(1.0), DAC(0.9), REM(1.1)	DEX(20548), DAC(16490), REM(20208)		R2(6.5), R3(1.2)	DEX(13868114), DAC(3299235), REM(11533490)		
13	47	22	OSTP (20) + DEX (20) + DAC (20) +REM (20)	10	25	73	27 5mM AA	0.03	239	Dilution with 30% methanol	OSTP(11.219), DEX(13.759), DAC(15.987), REM(17.344)	OSTP(1.051), DEX(1.101), DAC(1.152), REM(1.570)	OSTP(2.2), DEX(1.1), DAC(1.1), REM(1.1)	OSTP(6809), DEX(20322), DAC(19720), REM(20152)	0.721969697	R1(5.3), R2(5.2), R3(2.8)	OSTP(3959690), DEX(9970360), DAC(4765866), REM(23119152)	if concentration is 50 ug, AUC WOULD be OSTP(9899225), DEX(24925900), DAC(11914665), REM(57797880)	AUC responses of OSTP, DAC & REM increased by increasing methanol% in the solvent because their solubility increased and therfor their adsorbtion during filtration diminished.
13	48	23	OSTP (20) + DEX (20) + DAC (20) +REM (20)	10	25	73	27 5mM AA	0.07	239	Dilution with 30% methanol	OSTP(4.914), DEX(6.066), DAC(7.107), REM(7.677)	OSTP(0.590), DEX(0.800), DAC(0.533), REM(0.709)	OSTP(2.0), DEX(1.2), DAC(1.2), REM(1.2)	OSTP(5359), DEX(16970), DAC(14967), REM(17758)	0.741754386	R1(4.9), R2(4.8), R3(2.4)	OSTP(1820154), DEX(5366742), DAC(1996997), REM(9890204)	if concentration is 50 ug, AUC WOULD be OSTP(4550385), DEX(13416855), DAC(4992492.5), REM(24725510)	

Table S3: Physical characters of dexamesasone phosphate, dexamesasone, oseltamivir phosphate, daclatasivir dihydrochloride, and remdesivir

Drug	Water solubility	The log P (log Kow)	Acidic function group			Basic function group		
			**Pka (strongest acidic)**	**Status at pH 4.8 ± 0.5**	**Status at pH 7.5 ± 0.5**	**Pka (strongest basic)**	**Status at pH 4.8 ± 0.5**	**Status at pH 7.5 ± 0.5**
**Dexamesasone phosphate**	2.9 mg mL^{-1} [85]	1.64 [86]	1.89 [87]	Ionized	Ionized	-3.4 [85]	Unionized	Unionized
			6.18 [87]	Partially unionized (unionized molecules > ionized molecules)	Partially ionized (ionized molecules > unionized molecules)			
**Oseltamivir phosphate [8]**	0.686 mg mL^{-1}	1.30	14.03	Unionized	Unionized	9.31	Ionized	Partially ionized (ionized molecules > unionized molecules)
**Dexamesasone [10]**	0.0505 mg mL^{-1}	1.93	12.42	Unionized	Unionized	-3.3	Unionized	Unionized
**Daclatasivir dihydrochloride [11]**	0.00283 mg mL^{-1}	3.47	3.82	Partially ionized (ionized molecules > unionized molecules)	ionized	6.09	Ionized	Partially unionized (unionized molecules > ionized molecules)
**Remdesivir**	0.339 mg mL^{-1} [14]	3.20 [15]	10.23 [13]	Unionized	Unionized	0.65 [13]	Unionized	Unionized

Table S4: 2^3 FFD experimental factorial designs and their dependent responses at 239 nm for RPUPLC-PDA separation of the oseltamivir phosphate/dexamethasone/daclatasvir dihydrochloride/remdesivir mixture.

Design order		Experimental factorial design[a]			Dependent responses																Chromatograms
					OSTP				DEX				DAC				REM				
Std Order	Run Order	(A) MeOH %[b]	(B) AA conc.(mM)[c]	(C) FR (mL min^{-1})[d]	Rt_1[e]	k'[f]	T_1[g]	N_1[h]	Rt_2	R_{S1}	T_2	N_2	Rt_3	R_{S2}	T_3	N_3	Rt_4	R_{S3}	T_4	N_4	
1	1	73	5	0.03	11.219	0.6998	2.29915	6809.2	13.759	5.34438	1.13791	20322.7	15.987	5.21040	1.12746	19720.2	17.344	2.82716	1.14042	20152.9	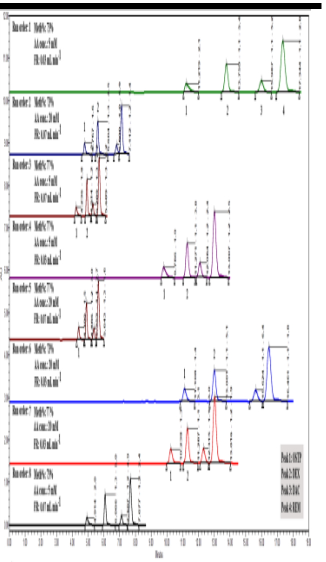
3	2	73	20	0.07	4.767	0.9068	1.61160	10092.7	5.604	4.47886	1.25462	16045.0	6.800	5.85916	1.02646	14445.9	7.112	1.38912	1.24608	16934.3	
8	3	77	5	0.07	4.236	0.5129	1.79638	5701.4	4.914	3.48243	1.20322	15484.1	5.289	2.20553	1.29041	14155.1	5.689	2.18833	1.27273	15541.5	
7	4	77	5	0.03	9.786	0.4827	1.92799	7739.2	11.271	3.80682	1.11489	19859.5	12.084	2.40178	1.15635	19401.4	13.007	2.52150	1.15841	19318.4	
6	5	77	20	0.07	4.390	0.5679	1.49277	10341.8	4.886	2.95075	1.23185	15248.8	5.352	2.70153	1.14362	13769.4	5.643	1.57824	1.26564	15459.8	
4	6	73	20	0.03	11.108	0.6830	1.43560	14216.9	13.001	5.06843	1.13411	20835.4	15.624	6.39200	1.06398	19562.6	16.461	1.82012	1.14304	20572.9	
5	7	77	20	0.03	10.238	0.5512	1.29928	14492.7	11.287	3.11688	1.13861	19693.8	12.315	3.00949	1.13219	19760.5	13.016	1.91034	1.16296	19467.5	
2	8	73	5	0.07	4.914	0.7550	2.02631	5359.8	6.066	4.96165	1.25860	16970.8	7.107	4.88872	1.22936	14967.6	7.677	2.42245	1.23146	17758.4	

[a] Three replicates of each experimental design were performed.
[b] Methanol% (v/v) (low level 73% and high level 77%).
[c] Ammonium acetate concentration. (mM) (low level 5 mM and high level 20 mM).
[d] Flow rate (mL min^{-1}) (low level 0.03 and high level 0.07).
[e] Peak retention time.
[f] Peak capacity factor.
[g] Peak tailing was evaluated at 5% (USP).
[h] A drug peak's theoretical plate count.

Table S5: Estimated Effects (coded unites) for $k'_{(OSTP)}$, $T_{1(OSTP)}$, and $R_{S3(REM)}$ versus methanol% (A), ammonium acetate conc. (B) and flow rate (C)

**Term**	$k'_{(OSTP)}$	$T_{1(OSTP)}$	$R_{S3(REM)}$
**Methanol% (A)**	-0.2325	-0.2141	-0.0651
**Ammonium acetate conc. (B)**	0.0646	-0.5526	-0.8154
**Flow rate (C)**	0.0815	-0.0087	-0.3752

Table S6: Application of the proposed method for the analysis of standared solutions of oseltamivir phosphate/dexamethasone/daclatasvir dihydrochloride/remdesivir mixture

Oseltamivir phosphate

Proposed method						Reported method[23]	
Conc.[a] taken, ng mL^{-1}	Conc. found, ng mL^{-1}	Found, %[b]	Conc. taken, µg mL^{-1}	Conc. found, µg mL^{-1}	Found, %[b]	Conc. taken, µg mL^{-1}	Found, %[b]
10.0	10.020	100.201	0.5	0.492	98.400	2.500	100.350
25.0	24.622	98.487	1.0	0.985	98.520	10.000	99.781
50.0	49.538	99.075	5.0	5.038	100.767	15.000	100.087
125.0	126.924	101.539	15.0	14.980	99.864		
250.0	248.626	99.450	25.0	25.002	100.010		
500.0	500.271	100.054	30.0	30.002	100.008		
**Mean ± SD, %**		**99.801 ± 1.060**	**Mean ± SD, %**		**99.595 ± 0.935**		**100.073 ± 0.285**
t [c]		**0.587 (2.447)**[d]	***t*** [c]		**1.150 (2.447)**		
F [e]		**13.875 (19.296)**	***F*** [d]		**10.789 (19.296)**		

Dexamethasone

Proposed method			Reported method[71]	
Conc. taken, ng mL^{-1}	Conc. found, ng mL^{-1}	Found, %[b]	Conc. taken, µg mL^{-1}	Found, %[b]
50.0	49.387	98.774	1.000	100.390
125.0	125.063	100.050	5.000	99.901
250.0	249.508	99.803	20.000	100.005
1000.0	1005.768	100.577		
2500.0	2492.798	99.712		
5000.0	5002.477	100.050		
**Mean ± SD, %**		**99.828 ± 0.597**	**Mean ± SD, %**	**100.099 ± 0.257**
t [c]		**0.949 (2.365)**		
F [d]		**5.396 (19.296)**		

Daclatasvir dihydrochloride

Proposed method						Reported method[50]	
Conc. taken, ng mL^{-1}	Conc. found, ng mL^{-1}	Found, %[b]	Conc. taken, µg mL^{-1}	Conc. found, µg mL^{-1}	Found, %[b]	Conc. taken, µg mL^{-1}	Found, %[b]
25.0	25.146	100.582	0.5	0.498	99.580	5.0	98.357
50.0	50.923	101.847	1.0	0.987	98.694	10.0	101.232
125.0	123.502	98.802	2.5	2.481	99.221	20.0	99.795
250.0	251.298	100.519	5.0	5.019	100.389		
500.0	498.636	99.727	12.5	12.533	100.265		
1000.0	1000.495	100.049	25.0	24.982	99.928		
**Mean ± SD, %**		**100.254 ± 1.014**	**Mean ± SD, %**		**99.680 ± 0.648**	**Mean ± SD, %**	**99.795 ± 1.438**
t [c]		**0.496 (3.182)**	***t*** [c]		**0.132 (4.303)**		
F [d]		**2.009 (5.786)**	***F*** [d]		**4.921 (5.786)**		

Remdesivir

Proposed method						Reported method[57]	
Conc. taken, ng mL^{-1}	Conc. found, ng mL^{-1}	Found, %[b]	Conc. taken, µg mL^{-1}	Conc. found, µg mL^{-1}	Found, %[b]	Conc. taken, µg mL^{-1}	Found, %[b]
10.0	9.861	98.609	0.5	0.509	101.723	2.5	101.942
25.0	24.862	99.447	1.0	1.002	100.202	5.0	98.786
50.0	50.844	101.689	5.0	5.035	100.698	15.0	100.081
125.0	123.548	98.838	10.0	9.876	98.755		
250.0	251.194	100.478	15.0	15.087	100.580		
500.0	499.691	99.938	30.0	29.992	99.973		
**Mean ± SD, %**		**99.833 ± 1.141**	**Mean ± SD, %**		**100.322 ± 0.976**	**Mean ± SD, %**	**100.270 ± 1.587**
t [c]		**0.425 (3.182)**	***t*** [c]		**0.052 (3.182)**		
F [d]		**1.934 (5.786)**	***F*** [d]		**2.645 (5.786)**		

[a] Conc.: Concentration.
[b] The average of three determinations.
[c] Student's *t*-test between proposed & reported methods assuming unequal variances.
[d] values in parentheses are critical values of *t* and *F*, where P=0.05.
[e] *F*-Test (Two-Sample assuming unequal Variances) between proposed & reported methods.

Table S7: Accuracy and precision results for the determination of oseltamivir phosphate/dexamethasone/daclatasvir dihydrochloride/remdesivir mixtures by the proposed method.

**Drug name**		**Oseltamivir phosphate**						**Dexamethasone**			**Daclatasvir dihydrochloride**						**Remdesivir**					
**Linearity range**		**10-500 (ng mL^{-1})**			**0.5-30 (μg mL^{-1})**			**50-5000 (ng mL^{-1})**			**25-1000 (ng mL^{-1})**			**0.5-25 (μg mL^{-1})**			**10-500 (ng mL^{-1})**			**0.5-30 (μg mL^{-1})**		
**Conc. taken in mixture[a]**		**25.0**	**250.0**	**400.0**	**2.50**	**15.0**	**25.0**	**125.0**	**2500.0**	**4000.0**	**50.0**	**500.0**	**800.0**	**1.0**	**12.50**	**20.0**	**25.0**	**250.0**	**400.0**	**2.50**	**15.0**	**25.0**
**Intraday (repeatability)**	**Found, %**	98.44	99.59	99.73	99.52	99.18	99.59	100.09	99.34	98.30	101.99	100.27	100.85	98.84	100.68	99.79	100.87	99.89	101.13	98.84	101.20	99.00
		98.64	100.45	98.16	99.22	98.15	99.81	100.37	100.44	98.85	102.15	99.70	100.28	98.49	99.87	99.76	99.64	100.50	101.48	99.15	102.24	101.19
		98.39	98.31	98.63	99.13	100.59	100.06	99.69	99.36	98.10	101.40	99.22	99.80	98.75	100.24	99.79	97.83	101.04	101.85	98.82	102.06	101.05
	**Mean, %**	**98.49**	**99.45**	**98.84**	**99.29**	**99.31**	**99.82**	**100.05**	**99.71**	**98.42**	**101.85**	**99.73**	**100.31**	**98.69**	**100.27**	**99.78**	**99.45**	**100.48**	**101.48**	**98.94**	**101.83**	**100.41**
	**SD, %**	**0.13**	**1.08**	**0.80**	**0.20**	**1.23**	**0.23**	**0.34**	**0.63**	**0.39**	**0.39**	**0.53**	**0.53**	**0.18**	**0.40**	**0.02**	**1.53**	**0.58**	**0.36**	**0.19**	**0.56**	**1.22**
	**RSD, %**	**0.13**	**1.08**	**0.81**	**0.20**	**1.23**	**0.23**	**0.34**	**0.64**	**0.39**	**0.39**	**0.53**	**0.52**	**0.18**	**0.40**	**0.02**	**1.54**	**0.57**	**0.36**	**0.19**	**0.55**	**1.22**
**Interday (intermediate percision) b**	**Found, %**	98.49	99.45	98.84	99.29	99.31	99.82	100.05	99.71	98.42	101.85	99.73	100.31	98.69	100.27	99.78	99.45	100.48	101.48	98.94	101.83	100.41
		101.23	98.08	100.51	99.21	100.34	100.00	100.13	101.88	99.89	102.07	98.85	100.50	101.58	99.65	99.66	101.60	101.67	100.25	102.17	98.65	98.65
		98.19	100.95	100.79	98.93	99.96	99.78	98.18	101.81	99.91	98.82	98.73	99.92	101.58	100.18	98.48	98.90	99.91	100.47	98.83	99.67	102.35
	**Mean, %**	**99.30**	**99.49**	**100.05**	**99.14**	**99.87**	**99.87**	**99.45**	**101.13**	**99.40**	**100.91**	**99.10**	**100.24**	**100.62**	**100.03**	**99.31**	**99.98**	**100.69**	**100.73**	**99.98**	**100.05**	**100.47**
	**SD, %**	**1.68**	**1.44**	**1.05**	**0.19**	**0.52**	**0.12**	**1.10**	**1.23**	**0.85**	**1.82**	**0.54**	**0.30**	**1.67**	**0.34**	**0.72**	**1.43**	**0.90**	**0.66**	**1.90**	**1.63**	**1.85**
	**RSD, %**	**1.69**	**1.44**	**1.05**	**0.19**	**0.52**	**0.12**	**1.11**	**1.22**	**0.86**	**1.80**	**0.55**	**0.29**	**1.66**	**0.33**	**0.72**	**1.43**	**0.89**	**0.65**	**1.90**	**1.63**	**1.84**

[a] Each mixture contains equal concentrations of OSTP, DEX, DAC, and REM.
[b] Each result is the average of three determinations.

Table S8: The average content for oseltamivir phosphate found in OSELTAMIVIR hard gelatin capsules, dexamethasone in DEXAZONE tablets, daclatasvir dihydrochloride JAVIDACLA film coated tablets, and remdesivir in REMDESIVIR vials.

	**Oseltamivir phosphate in OSELTAMIVIR capsules**			**Dexamethasone in DEXAZONE tablets**		**Daclatasvir dihydrochloride in JAVIDACLA tablets**			**Remdesivir in REMDESIVIR vails**		
	**Proposed method**		**Reference method[23]**	**Proposed method**	**Reference method[71]**	**Proposed method**		**Reference method[50]**	**Proposed method**		**Reference method[75]**
**Predicted concentration**	**246.25 (ng mL^{-1})[a]**	**24.625 (µg mL^{-1})[b]**		**2500.000 (ng mL^{-1})**		**164.800 (ng mL^{-1})[a]**	**16.48 (µg mL^{-1})[b]**		**125.000 (ng mL^{-1})[a]**	**12.500 (µg mL^{-1})[b]**	
**Found, %**	91.243	93.020	93.959	102.777	100.735	97.258	98.983	99.106	90.646	92.827	91.863
	93.574	94.784	91.679	100.049	102.959	99.097	97.250	98.106	91.539	96.962	93.501
	92.731	91.669	94.934	101.966	100.811	97.789	97.845	99.458	91.112	93.422	92.214
**Average content[c] ± SD, %**	92.516 ± 1.180	93.158 ± 1.562	93.524 ± 1.671	101.597 ± 1.401	101.501 ± 1.262	98.048 ± 0.947	98.026 ± 0.880	98.890 ± 0.702	91.099 ± 0.447	94.403 ± 2.236	92.526 ± 0.862
**ANOVA-F[d]**	0.322					0.001			6.300		
***t*[e]**	**0.854**	**0.278**		**0.088**		**1.237**	**1.329**		**2.545**	**1.357**	
***F*[f]**	**2.004**	**1.145**		**1.231**		**1.820**	**1.575**		**3.720**	**6.727**	

[a] It was determined by the regression equation of the low linearity range.
[b] It was determined by the regression equation of the high linearity range.
[c] The BP range for five dosage forms is 88.4–111.8%, as the weight of active ingredients in capsule/tablet is less than 0.12 g.
[d] Calculated F, ANOVA test for equivalence study between proposed methods (grouped by column), ANOVA-F-critical = 7.709 (alpha = 0.05).
[e] Student's *t*-test between assuming equal variances, *t*-test-critical = 2.776 (alpha = 0.05).
[f] *F*-Test (Two-Sample assuming unequal Variances) between proposed & reported methods, *F*-Test-critical = 19.000 (alpha = 0.05).

Table S9: Classic standard addition method

Fist approach: for low linearity ranges of OSTP, DAC, and REM				Second approach: for DEX and high linearity ranges of OSTP, DAC, and REM.				
**Drug name**	**OSTP**	**DAC**	**REM**	**Drug name**	**OSTP**	**DEX**	**DAC**	**REM**
**Standard nominal concentration[a], ng mL^{-1}**	**% Recovery**	**% Recovery**	**% Recovery**	**Standard nominal concentration[b], µg mL^{-1}**	**% Recovery**	**% Recovery**	**% Recovery**	**% Recovery**
0.000[c]	-	-	-	0.000[c]	-	-	-	-
0.250	98.682	91.680	102.662	0.500	96.938	92.793	93.304	94.262
2.500	95.703	91.542	109.058	5.000	105.666	98.845	108.551	99.329
6.250	98.365	101.131	100.022	12.500	97.109	101.636	99.185	104.609
12.500	100.581	100.059	99.631	25.000	100.497	99.640	99.864	98.877
**Intercept (a)**	17955.685	18646.511	78666.834	**Intercept (a)**	1928899.050	740734.699	1820281.078	7907084.743
**Slope (b)**	78.290	115.571	678.597	**Slope (b)**	84920.227	287949.857	113227.789	664481.429
**Coefficient of determination (R^2)**	0.999570	0.999323	0.999186	**Coefficient of determination (R^2)**	0.999479	0.999874	0.998574	0.998932
**Predicted average content, ηg mL^{-1}**	246.250	164.800	125.000	**Predicted average content, µg mL^{-1}**	24.625	2.500	16.480	12.500
**Average content (Found); ηg mL^{-1}**	229.348	161.342	115.926	**Average content (Found);µg mL^{-1}**	22.714	2.572	16.076	11.900
**Average content; % (by Traditional standard addition)**	93.114	99.049	92.593	**Average content; % (by Traditional standard addition)**	92.219	102.185	98.693	95.045
**Average content[d]; % (by proposed method)**	92.516	98.048	91.099	**Average content[d]; % (by proposed method)**	93.158	101.597	98.026	94.403

[a] Standard concentrations after dilution (original standard concentrations were 250 ηg mL^{-1} per each drug).
[b] Standard concentrations after dilution (original standard concentrations were 500 µg mL^{-1} per each drug).
[c] Its response was the average of three measurements.
[d] The average of the same three samples used in the traditional standard addition method.

Table S10: Estimated robustness of the proposed method.

A – Change in flow rate (mL min^{-1})[a]									
System suitability parameter		Flow rate (mL min^{-1})			Robustness			ANOVA (single factor) (Grouped by columns)	
		0.040	0.480	0.050	Mean	SD	RSD,%	F[b] (F-critical = 9.552)	P-Value[c] (Alpha = 0.05)
$k'_{(OSTP)}$	Practical	0.513	0.526	0.572	0.537	0.031	5.772	**0.547**	**0.627**
	Factorial design value	0.574	0.584	0.586	0.581	0.006	1.093		
$T_{1(OSTP)}$	Practical	1.588	1.633	1.530	1.584	0.052	3.274	**0.041**	**0.960**
	Factorial design value	1.874	1.854	1.849	1.859	0.013	0.712		
$N_{1(OSTP)}$	Practical	8751.740	8636.185	8648.500	8678.808	63.460	0.731	**0.259**	**0.787**
	Factorial design value	8331.212	7864.513	7747.838	7981.188	308.692	3.868		
$R_{S1(DEX)}$	Practical	4.261	4.204	4.150	4.205	0.055	1.317	**0.426**	**0.687**
	Factorial design value	4.105	4.038	4.021	4.055	0.044	1.093		
$T_{2(DEX)}$	Practical	1.177	1.202	1.220	1.200	0.021	1.780	**0.949**	**0.479**
	Factorial design value	1.150	1.170	1.175	1.165	0.013	1.130		
$N_{2(DEX)}$	Practical	18076.620	17246.684	17107.280	17476.861	524.062	2.999	**1.471**	**0.359**
	Factorial design value	18983.679	18153.300	17945.706	18360.895	549.244	2.991		
$R_{S2(DAC)}$	Practical	3.505	3.462	3.430	3.466	0.038	1.087	**0.651**	**0.582**
	Factorial design value	3.416	3.363	3.349	3.376	0.035	1.048		
$T_{3(DAC)}$	Practical	1.159	1.156	1.150	1.155	0.004	0.380	**0.120**	**0.891**
	Factorial design value	1.163	1.183	1.187	1.178	0.013	1.083		
$N_{3(DAC)}$	Practical	17506.590	17272.104	17174.520	17317.738	170.674	0.986	**3.690**	**0.155**
	Factorial design value	18241.150	17197.624	16936.742	17458.505	690.228	3.954		
$R_{S3(REM)}$	Practical	2.238	2.138	2.120	2.165	0.063	2.924	**0.503**	**0.648**
	Factorial design value	2.374	2.303	2.285	2.321	0.047	2.041		
$T_{4(REM)}$	Practical	1.198	1.215	1.220	1.211	0.011	0.944	**2.720**	**0.212**
	Factorial design value	1.180	1.201	1.206	1.196	0.014	1.172		
$N_{4(REM)}$	Practical	19115.900	19322.030	19315.300	19251.077	117.115	0.608	**0.098**	**0.909**
	Factorial design value	18778.379	18089.100	17916.780	18261.420	455.915	2.497		

[a] The mobile phase contained 75.7% (v/v) methanol and 24.3% (v/v) ammonium acetate (8.1818 mM).
[b] When ANOVA-F is smaller than ANOVA-F-critical, the null hypothesis is accepted (no difference between practical and factorial design values).
[c] When the P-Value is greater than the significance level (0.05), the null hypothesis is accepted (no difference between practical and factorial design values).

B – Change in methanol% in mobile phase[d]									
**System suitability parameter**		**Methanol%**			**Robustness**			**ANOVA (single factor) (Grouped by columns)**	
		**75.0**	**75.7**	**76.0**	**Mean**	**SD**	**RSD,%**	**F[b] (F-critical = 9.552)**	**P-Value[c] (Alpha = 0.05)**
$k'_{(OSTP)}$	**Practical**	0.563	0.526	0.517	0.635	0.015	2.384	**0.825**	**0.518**
	**Factorial design value**	0.623	0.584	0.566	0.629	0.020	3.132		
$T_{1(OSTP)}$	**Practical**	1.553	1.633	1.536	1.236	0.019	1.557	**0.018**	**0.982**
	**Factorial design value**	1.901	1.854	1.834	0.966	0.027	2.796		
$N_{1(OSTP)}$	**Practical**	8819.420	8636.185	8653.110	13053.192	523.744	4.012	**0.002**	**0.998**
	**Factorial design value**	7762.994	7864.513	7908.021	10752.220	293.313	2.728		
$R_{S1(DEX)}$	**Practical**	4.387	4.204	4.338	1.988	0.098	4.918	**0.973**	**0.472**
	**Factorial design value**	4.312	4.038	3.921	1.824	0.148	8.123		
$T_{2(DEX)}$	**Practical**	1.203	1.202	1.194	1.198	0.006	0.467	**0.094**	**0.913**
	**Factorial design value**	1.176	1.170	1.168	1.088	0.011	1.055		
$N_{2(DEX)}$	**Practical**	17393.404	17246.684	19057.124	20680.555	962.415	4.654	**0.985**	**0.469**
	**Factorial design value**	18317.277	18153.300	18083.025	21069.553	221.894	1.053		
$R_{S2(DAC)}$	**Practical**	3.989	3.462	3.863	4.263	0.285	6.687	**1.678**	**0.324**
	**Factorial design value**	3.864	3.363	3.148	4.419	0.438	9.920		
$T_{3(DAC)}$	**Practical**	1.147	1.156	1.143	1.124	0.008	0.732	**0.073**	**0.931**
	**Factorial design value**	1.173	1.183	1.187	1.037	0.017	1.655		
$N_{3(DAC)}$	**Practical**	17385.194	17272.104	19614.094	18510.115	1284.715	6.941	**0.815**	**0.522**
	**Factorial design value**	17279.474	17197.624	17162.545	18424.492	59.001	0.320		
$R_{S3(REM)}$	**Practical**	2.089	2.138	2.099	1.594	0.129	8.080	**0.018**	**0.982**
	**Factorial design value**	2.335	2.303	2.289	1.681	0.037	2.219		
$T_{4(REM)}$	**Practical**	1.202	1.215	1.204	1.207	0.007	0.594	**1.083**	**0.443**
	**Factorial design value**	1.196	1.201	1.203	1.100	0.011	1.003		
$N_{4(REM)}$	**Practical**	19525.970	19322.030	21807.850	20218.617	1380.089	6.826	**0.273**	**0.778**
	**Factorial design value**	18337.133	18089.100	17982.800	20288.845	197.471	0.973		

[d] The flow rate was 0.048 mL min^{-1} and ammonium acetate was 8.1818 mM.

C – Change in ammonium acetate concentration (mM)[e]

System suitability parameter		Ammonium acetate (mM)			Robustness			ANOVA (single factor) (Grouped by columns)	
		8.0000	8.1818	8.4000	Mean	SD	RSD,%	F[b] (F-critical = 9.552)	P-Value[c] (Alpha = 0.05)
$k'_{(OSTP)}$	Practical	0.434	0.526	0.542	0.647	0.039	6.054	0.309	0.755
	Factorial design value	0.583	0.584	0.584	0.624	0.003	0.476		
$T_{1(OSTP)}$	Practical	1.593	1.633	1.566	1.291	0.076	5.859	0.008	0.992
	Factorial design value	1.861	1.854	1.846	0.959	0.004	0.461		
$N_{1(OSTP)}$	Practical	8423.100	8636.185	8486.430	11865.525	712.162	6.002	0.052	0.950
	Factorial design value	7792.604	7864.513	7950.819	10681.654	93.175	0.872		
$R_{S1(DEX)}$	Practical	4.259	4.204	4.770	2.200	0.378	17.177	0.453	0.673
	Factorial design value	4.045	4.038	4.030	1.786	0.010	0.545		
$T_{2(DEX)}$	Practical	1.183	1.202	1.163	1.201	0.010	0.831	0.930	0.485
	Factorial design value	1.170	1.170	1.171	1.088	0.011	1.019		
$N_{2(DEX)}$	Practical	15481.090	17246.684	17069.190	18776.061	1410.156	7.510	0.309	0.755
	Factorial design value	18155.442	18153.300	18150.730	21012.696	13.148	0.063		
$R_{S2(DAC)}$	Practical	4.166	3.462	5.402	4.835	0.990	20.477	0.613	0.598
	Factorial design value	3.354	3.363	3.373	4.306	0.011	0.252		
$T_{3(DAC)}$	Practical	1.138	1.156	1.068	1.096	0.048	4.341	0.432	0.684
	Factorial design value	1.184	1.183	1.181	1.040	0.010	0.954		
$N_{3(DAC)}$	Practical	15238.000	17272.104	19916.884	17768.821	2529.182	14.234	1.466	0.360
	Factorial design value	17198.693	17197.624	17196.340	18410.321	19.127	0.104		
$R_{S3(REM)}$	Practical	2.066	2.138	1.932	1.594	0.129	8.080	0.172	0.850
	Factorial design value	2.312	2.303	2.292	1.691	0.009	0.517		
$T_{4(REM)}$	Practical	1.220	1.215	1.198	1.211	0.011	0.941	0.695	0.565
	Factorial design value	1.201	1.201	1.201	1.100	0.011	1.011		
$N_{4(REM)}$	Practical	17961.550	19322.030	22979.460	20087.680	2595.095	12.919	0.794	0.529
	Factorial design value	18089.282	18089.100	18088.881	20238.324	13.009	0.064		

[e] The flow rate was 0.048 mL min^{-1} and the methanol to ammonium acetate ratio was 75.7:24.3.

Table S11: The uniformity of dosage unit for oseltamivir phosphate in OSELTAMIVIR capsules and daclatasivir dihydrochloride in JAVIDACLA tablets.

OSELTAMIVIR capsule (98.5 mg OSTP)	Capsule content mass (Wi) , g	Nominal amount; 246.25 ($ng\ mL^{-1}$) [a]		Nominal amount; 24.625 ($\mu g\ mL^{-1}$)[a]		JAVIDACLA tablet (60 mg DAC)	Nominal amount; 164.800 ($ng\ mL^{-1}$)[a]		Nominal amount; 16.480 ($\mu g\ mL^{-1}$)[a]	
		Amount (mg)/Capsule	A, % found per capsule	Amount (mg)/Capsule	A, % found per capsule		Amount (mg)/Tablet	A, % found per tablet	Amount (mg)/Tablet	A, % found per tablet
**Capsule 1**	0.239	90.940	92.303	90.392	91.747	Tablet 1	64.891	99.592	64.473	98.950
**Capsule 2**	0.230	91.413	92.783	91.697	93.071	Tablet 2	66.068	101.398	64.693	99.288
**Capsule 3**	0.235	89.582	90.924	90.220	91.572	Tablet 3	66.015	101.317	65.692	100.822
**Capsule 4**	0.228	90.771	92.131	89.040	90.374	Tablet 4	64.879	99.574	66.033	101.345
**Capsule 5**	0.238	92.483	93.869	89.693	91.037	Tablet 5	65.460	100.466	65.826	101.027
**Capsule 6**	0.237	90.303	91.657	92.469	93.855	Tablet 6	66.140	101.509	65.516	100.551
**Capsule 7**	0.239	91.015	92.379	92.117	93.497	Tablet 7	65.619	100.709	65.511	100.543
**Capsule 8**	0.238	90.697	92.056	91.999	93.378	Tablet 8	64.887	99.586	65.353	100.302
**Capsule 9**	0.233	88.731	90.061	91.320	92.689	Tablet 9	66.502	102.064	65.116	99.938
**Capsule 10**	0.236	90.756	92.116	91.062	92.427	Tablet 10	65.245	100.136	63.931	98.119
**Mean**	0.235	90.669	92.028	91.001	92.365	**Mean**	65.571	100.635	65.214	100.088
**SD**	0.004	1.007	1.022	1.132	1.149	**SD**	0.593	0.910	0.663	1.017
**AV (Acceptance value), %[b]**		**11.130**		**11.308**		**AV (Acceptance value), %[b]**	**3.049**		**3.852**	
**F-ANOVA[c]**		**0.479**				**F-ANOVA**	**1.606**			

[a] Each result is the average of three separate determinations.
[b] The requirements for content uniformity are met if the acceptance value (AV) of the first 10 dosage units is less than or equal to L1 (15.000).
[c] Calculated F, ANOVA test for equivalence study between proposed methods (grouped by column), ANOVA-F-critical = 4.414 (alpha = 0.05).

Table S12: The uniformity of dosage unit for dexamethasone in DEXAZONE tablet and remdesivir in REMDESIVIR vails.

DEXAZONE tablet (0.5 mg DEX)	Nominal amount; 2500.000 (ng mL^{-1}) [a]		REMDESIVIR vial (100 mg REM)	Nominal amount; 125.000 (ng mL^{-1})[a]		Nominal amount; 12.500 (µg mL^{-1})[a]	
	**Amount (mg)/Tablet**	**A, % found per tablet**		**Amount (mg)/Vail**	**A, % found per Vail**	**Amount (mg)/Vail**	**A, % found per Vail**
**Tablet 1**	0.517	102.663	**Vail 1**	93.191	93.043	94.774	94.622
**Tablet 2**	0.497	98.766	**Vail 2**	93.723	93.573	95.204	95.052
**Tablet 3**	0.502	99.641	**Vail 3**	99.157	98.999	99.064	98.906
**Tablet 4**	0.506	100.402	**Vail 4**	96.765	96.611	99.646	99.487
**Tablet 5**	0.522	103.603	**Vail 5**	93.534	93.385	100.642	100.482
**Tablet 6**	0.501	99.431	**Vail 6**	93.775	93.626	100.742	100.582
**Tablet 7**	0.514	102.053	**Vail 7**	96.386	96.233	100.251	100.091
**Tablet 8**	0.522	103.774	**Vail 8**	93.472	93.323	96.199	96.046
**Tablet 9**	0.508	100.819	**Vail 9**	100.159	100.000	95.342	95.190
**Tablet 10**	0.523	103.876	**Vail 10**	93.222	93.073	95.351	95.199
**Mean**	0.511	101.503	**Mean**	95.338	95.187	97.722	97.566
**SD**	0.010	1.937	**SD**	2.621	2.617	2.543	2.539
**AV (Acceptance value), %**[b]	**4.653**		**AV (Acceptance value), %**[b]	**9.851**		**7.027**	
			**F-ANOVA**[c]	**4.258**			

[a] Each result is the average of three separate determinations.
[b] The requirements for content uniformity are met if the acceptance value (AV) of the first 10 dosage units is less than or equal to L1 (15.000).
[c] Calculated F, ANOVA test for equivalence study between proposed methods (grouped by column), ANOVA-F-critical = 4.414 (alpha = 0.05).

Table S13: The penalty points of the proposed method according to the analytical Eco-Scale per sample

	**Proposed method for quaternary mixture**	**Proposed method for oseltamivir phosphate alone**	**Proposed method for dexamethasone alone**	**Proposed method for daclatasivir dihydrochloride alone**	**Proposed method for remdesivir alone**
**Reagents**	**Penalty points**	**Penalty points**	**Penalty points**	**Penalty points**	**Penalty points**
Methanol (≈ 23 mL)	12	12	12	12	12
Ammonium acetate 0.0007 gm)	1	1	1	1	1
OSTP (0.010 gm)	1	1	-	-	-
DEX (0.010 gm)	2	-	2	-	-
DAC (0.010 gm)	0	-	-	0	-
REM (0.010 gm)	1	-	-	-	1
**Instrument**	**Penalty points**	**Penalty points**	**Penalty points**	**Penalty points**	**Penalty points**
LC[a] (≤ 1.5 kWh per sample)	1	1	1	1	1
Occupational hazard (analytical process hermetization)	0	0	0	0	0
Waste (less than1 mL, no treatment)	4	4	4	4	4
**Total penalty points**	22	19	20	18	19
**Analytical eco-scale total score [b]**	78	81	80	82	81

[a] Liquid chromatography.
[b] If the score is more than 75, it represents excellent green analysis.
If the score is more than 50, it represents acceptable green analysis.
If the score is less than 50, it represents inadequate green analysis.

Supplementary material Equations:

- **Equation S1:**

  $\boldsymbol{k'}_{\mathbf{(OSTP)}} = 5.67578 - (0.0680771 \times \text{MeOH\%}) - (0.279498 \times \text{AA conc.}) - (16.3131 \times \text{FR}) + (0.00369792 \times \text{MeOH\%} \times \text{AA conc.}) + (0.223125 \times \text{MeOH\%} \times \text{FR}) + (5.81988 \times \text{AA conc.} \times \text{FR}) - (0.075875 \times \text{MeOH\%} \times \text{AA conc.} \times \text{FR})$

- **Equation S2:**

  $\mathbf{R}_{S1(DEX)} = 30.3466 - (0.3380 \times \text{MeOH\%}) + (0.828765 \times \text{AA conc.}) + (21.063 \times \text{FR}) - (0.0115 \times \text{MeOH\%} \times \text{AA conc.}) - (0.3960 \times \text{MeOH\%} \times \text{FR}) - (11.4495 \times \text{AA conc.} \times \text{FR}) + (0.1521 \times \text{MeOH\%} \times \text{AA conc.} \times \text{FR})$

- **Equation S3:**

  $\mathbf{R}_{S2(DAC)} = 54.0344 - (0.6716 \times \text{MeOH\%}) + (0.87831 \times \text{AA conc.}) - (48.3867 \times \text{FR}) - (0.0108 \times \text{MeOH\%} \times \text{AA conc.}) + (0.5768 \times \text{MeOH\%} \times \text{FR}) - (3.3769 \times \text{AA conc.} \times \text{FR}) + (0.0414 \times \text{MeOH\%} \times \text{AA conc.} \times \text{FR})$

- **Equation S4:**

  $\mathbf{R}_{S3(REM)} = 12.301 - (0.1211 \times \text{MeOH\%}) - (0.522523 \times \text{AA conc.}) - (38.3824 \times \text{FR}) + (0.0063 \times \text{MeOH\%} \times \text{AA conc.}) + (0.3902 \times \text{MeOH\%} \times \text{FR}) - (0.8759 \times \text{AA conc.} \times \text{FR}) + (0.0114 \times \text{MeOH\%} \times \text{AA conc.} \times \text{FR})$

- **Equation S5:**

  $\mathbf{T}_{\mathbf{1(OSTP)}} = 13.6038 - (0.1466 \times \text{MeOH\%}) - (0.4786 \times \text{AA conc.}) - (93.8181 \times \text{FR}) + (0.0055 \times \text{MeOH\%} \times \text{AA conc.}) + (1.1405 \times \text{MeOH\%} \times \text{FR}) + (4.5118 \times \text{AA conc.} \times \text{FR}) - (0.0516 \times \text{MeOH\%} \times \text{AA conc.} \times \text{FR})$

- **Equation S6:**

  $\mathbf{T}_{\mathbf{2(DEX)}} = 1.1702 - (0.0017 \times \text{MeOH\%}) - (0.0291 \times \text{AA conc.}) + (18.5519 \times \text{FR}) + (0.0004 \times \text{MeOH\%} \times \text{AA conc.}) - (0.2128 \times \text{MeOH\%} \times \text{FR}) - (0.1546 \times \text{AA conc.} \times \text{FR}) + (0.0021 \times \text{MeOH\%} \times \text{AA conc.} \times \text{FR})$

- **Equation S7:**

$T_{3(DAC)} = 1.1129 - (0.001 \times MeOH\%) - (0.0298 \times AA\ conc.) - (8.4104 \times FR) + (0.0004 \times MeOH\% \times AA\ conc.) + (0.166 \times MeOH\% \times FR) - (0.7429 \times AA\ conc. \times FR) + (0.007 \times MeOH\% \times AA\ conc. \times FR)$

- **Equation S8:**

  $T_{4(REM)} = 1.184 - (0.0015 \times MeOH\%) - (0.0243 \times AA\ conc.) - (12.0365 \times FR) + (0.0003 \times MeOH\% \times AA\ conc.) + (0.1947 \times MeOH\% \times FR) + (0.7388 \times AA\ conc. \times FR) - (0.0098 \times MeOH\% \times AA\ conc. \times FR)$

- **Equation S9:**

  $N_{1(OSTP)} = -26811.8 + (432.466 \times MeOH\%) + (1936.22 \times AA\ conc.) + (339932 \times FR) - (17.9264 \times MeOH\% \times AA\ conc.) - (4847.62 \times MeOH\% \times FR) - (21543.7 \times AA\ conc. \times FR) + (234.05 \times MeOH\% \times AA\ conc. \times FR)$

- **Equation S10:**

  $N_{2(DEX)} = 6380.98 + (218.1540 \times MeOH\%) + (2180.45 \times AA\ conc.) + (603277 \times FR) - (28.4158 \times MeOH\% \times AA\ conc.) - (9247.81 \times MeOH\% \times FR) - (44031 \times AA\ conc. \times FR) + (570.325 \times MeOH\% \times AA\ conc. \times FR)$

- **Equation S11:**

  $N_{3(DAC)} = 27185.7 - (53.9665 \times MeOH\%) - (968.175 \times AA\ conc.) + (51600.2 \times FR) + (13.3682 \times MeOH\% \times AA\ conc.) - (2292.9 \times MeOH\% \times FR) + (10969.7 \times AA\ conc. \times FR) - (158.583 \times MeOH\% \times AA\ conc. \times FR)$

- **Equation S12:**

  $N_{4(REM)} = 11534 + (136.491 \times MeOH\%) + (1344.46 \times AA\ conc.) + (735351 \times FR) - (17.1817 \times MeOH\% \times AA\ conc.) - (10751.3 \times MeOH\% \times FR) - (32895.3 \times AA\ conc. \times FR) + (422.217 \times MeOH\% \times AA\ conc. \times FR)$
